# Selective Constraints on Coding Sequences of Nervous System Genes Are a Major Determinant of Duplicate Gene Retention in Vertebrates

**DOI:** 10.1093/molbev/msx199

**Published:** 2017-07-16

**Authors:** Julien Roux, Jialin Liu, Marc Robinson-Rechavi

**Affiliations:** 1 Département d’Ecologie et d’Evolution, Université de Lausanne, Lausanne, Switzerland; 2 Swiss Institute of Bioinformatics, Lausanne, Switzerland

**Keywords:** whole-genome duplication, small-scale duplication, neuron, anatomy, gene expression, protein misfolding, protein interaction, translational accuracy

## Abstract

The evolutionary history of vertebrates is marked by three ancient whole-genome duplications: two successive rounds in the ancestor of vertebrates, and a third one specific to teleost fishes. Biased loss of most duplicates enriched the genome for specific genes, such as slow evolving genes, but this selective retention process is not well understood. To understand what drives the long-term preservation of duplicate genes, we characterized duplicated genes in terms of their expression patterns. We used a new method of expression enrichment analysis, TopAnat, applied to in situ hybridization data from thousands of genes from zebrafish and mouse. We showed that the presence of expression in the nervous system is a good predictor of a higher rate of retention of duplicate genes after whole-genome duplication. Further analyses suggest that purifying selection against the toxic effects of misfolded or misinteracting proteins, which is particularly strong in nonrenewing neural tissues, likely constrains the evolution of coding sequences of nervous system genes, leading indirectly to the preservation of duplicate genes after whole-genome duplication. Whole-genome duplications thus greatly contributed to the expansion of the toolkit of genes available for the evolution of profound novelties of the nervous system at the base of the vertebrate radiation.

## Introduction

The process of gene duplication plays a major role in the evolution of genomes, as it provides raw material for innovation (Lynch and Conery 2000; Conant and Wolfe 2008; Van de Peer etal. 2009). But only a minority of the gene duplication events reach fixation in a species, and survive in the long term with two functional gene copies (Innan and Kondrashov 2010). It is not yet clear what factors drive this process of selective retention, but it is clear that it is nonrandom (Davis and Petrov 2004).

Focusing on whole-genome duplication events allows quantification of the long-term retention bias alone, the whole gene set having been fixed in duplicate (Singh etal. 2012). In vertebrates, it has been estimated that only 10–20% of the duplicates (or “ohnologs”) that originated from the ancient whole-genome duplications at the origin of the lineage (“2R” hypothesis; Ohno 1970; Holland etal. 1994; Hughes 1999; Putnam etal. 2008) or in teleost fishes (“3R” hypothesis; Jaillon etal. 2004; Meyer and Van de Peer 2005) were eventually retained in the long term (Brunet etal. 2006; Nakatani etal. 2007; Putnam etal. 2008; Smith and Keinath 2015). Retained genes do not constitute a random subset of genes. For instance, their protein sequences tend to be under strong selective constraint (Davis and Petrov 2004; Brunet etal. 2006; Howe etal. 2013). They tend to be involved in functions such as signaling, cognition and behavior, or regulation of transcription (Brunet etal. 2006; Putnam etal. 2008; Kassahn etal. 2009; Huminiecki and Heldin 2010; Schartl etal. 2013), and to be expressed late in development (Roux and Robinson-Rechavi 2008). The causal mechanisms linking such properties to increased retention after whole-genome duplication have not been fully clarified so far.

An interesting study found that in yeast and *Paramecium*, the expression level of genes was a major determinant of their duplication retention rate after whole-genome duplication (Gout etal. 2010), highly expressed genes being more retained, an effect that could not be explained indirectly by other factors. This observation is noteworthy since gene expression level is also known to be a major determinant of the rate of protein evolution across a wide range of species (Drummond etal. 2005; Gu and Su 2007; Drummond and Wilke 2008), highly expressed genes having lower rates of protein evolution.

The generalization of this result to vertebrates is complicated by their complex anatomy. One way to address this complexity is to investigate whether patterns of expression over anatomy could be linked to ohnolog retention rates. But surprisingly this question has rarely been addressed. On the basis of EST data, a study found little association between expression breadth—the number of tissues in which a gene is expressed—and retention rate after the 2R whole-genome duplication (Satake etal. 2012). However, the authors observed lower retention of the fast-evolving genes expressed in endodermal tissues, such as the digestive tract, compared with slow-evolving genes expressed in ectodermal tissues, such as the nervous system. The expression patterns were opposite for small-scale duplication events, in agreement with other results showing that these two types of duplications tend to affect opposite sets of genes (Davis and Petrov 2005; Makino etal. 2009).

These observations suggest that anatomical expression patterns of genes might help to understand the process of ohnolog retention in vertebrates. Unfortunately the techniques used to study gene expression patterns on a genomic scale, previously ESTs and microarrays, and more recently RNA-seq, usually lack anatomical precision. In this paper, we took advantage of bioinformatics integration of another source of expression data, in situ hybridizations. Expression patterns obtained with this technique are very precise, sometimes down to the cellular resolution (Lein etal. 2007; Diez-Roux etal. 2011; Jacobs etal. 2011). They are also very inclusive, since it is possible to visualize the expression of a particular gene in the entirety of anatomical structures present in a histological section or even an entire organism (“whole-mount” in situ hybridizations), without selecting a priori a tissue to dissect. Compared with other techniques, there is also less averaging or dilution of the expression signal for genes whose expression is heterogeneous among the cells or substructures of a tissue (Altschuler and Wu 2010; Pantalacci and Semon 2015).

A drawback of in situ hybridizations, however, is that they usually give information on only one, or sometimes a handful of genes. Fortunately, there have been several efforts to generate with this technique high-throughput atlases of gene expression patterns in model organisms, notably zebrafish and mouse (e.g., Neidhardt etal. 2000; Thisse etal. 2004; Lein etal. 2007; Diez-Roux etal. 2011). Thus, there are thousands of in situ hybridizations publicly available, allowing us to perform analyses at the genomic scale. Even more valuable, the expression patterns revealed by these hybridizations have been manually annotated to terms from anatomical ontologies, notably the cross-species ontology Uberon describing anatomical structures and their relationships in animals (Hayamizu etal. 2005, 2013; Sprague etal. 2006; Bastian etal. 2008; Mungall etal. 2012; Haendel etal. 2014).

To detect the biases in anatomical expression patterns of ohnologs, we developed a novel bioinformatics approach. Similarly to the widely used functional enrichment tests performed on categories of the Gene Ontology (Ashburner etal. 2000; Yon Rhee etal. 2008), we used a Fisher’s exact test to detect an enrichment in the proportion of ohnologs expressed in each anatomical structure of the organism. This methodology allowed us to monitor expression biases with great precision, and to benefit from the information encoded in the ontology (e.g., parent–child relationships).

We observed that genes expressed in the nervous system had an increased chance of being retained after whole-genome duplication, whereas they had a decreased chance of being duplicated via small-scale duplication. This novel and robust observation helped us clarify the gene properties that causally influence the retention of duplicate genes. The rate of nonsynonymous substitutions of nervous-system genes, their level of optimization of synonymous codon usage at sites that are important for protein structure stability, and their maximum level of expression across neural tissues are significantly associated to retention rate, suggesting a major role of purifying selection on coding sequence on ohnolog retention in vertebrates. This selective force is particularly strong on nervous system genes, primarily preventing them from producing toxic protein products. It could have the unexpected consequence of lowering their probability of loss of function, leading to their evolutionary long-term retention.

This model is consistent with a model proposed to explain the counterintuitive expansion of human disease-causing genes after the 2R whole-genome duplication events (Singh etal. 2012; Malaguti etal. 2014), and is not exclusive of previously proposed models, for example, sub or neofunctionalization (Ohno 1970; Force etal. 1999; He and Zhang 2005), the dosage-balance hypothesis (Freeling and Thomas 2006; Makino and McLysaght 2010; Birchler and Veitia 2012; Singh etal. 2012), or selection for absolute dosage (Osborn etal. 2003; Innan and Kondrashov 2010). Rather it expands these models to illustrate the key role of anatomy in shaping the duplicated gene content of vertebrate genomes.

## Results

### 3R Ohnologs Are Biased for Nervous System Expression

Zebrafish 3R ohnologs were identified using a phylogenomics approach, and were used as input gene list in an expression enrichment test (see Materials and Methods, supplementary fig. S1, Supplementary Material online). The list of anatomical structures showing enrichment for expression of these genes is shown in table 1. At a false discovery rate (FDR) threshold of 10%, 25 structures were significantly enriched. The only significant depletion was for “unspecified,” a term indicating that the gene expression was assayed, but no anatomical structure was specified by the author.

**Table 1.** Zebrafish Anatomical Structures Showing a Significant Enrichment in Expression of Zebrafish 3R Ohnologs (FDR < 10%).

Organ ID	Organ Name	Number of Genes Expressed	Number of 3R Ohnologs Expressed	Number of 3R Ohnologs Expected	Enrichment Fold	*P*-Value	FDR
UBERON:2007001	*Dorso-rostral cluster*	18	9	1.88	4.79	2.91E-05	3.02E-03
UBERON:2007002	*Ventro-rostral cluster*	21	10	2.19	4.57	1.78E-05	2.00E-03
UBERON:2007003	*Ventro-caudal cluster*	19	9	1.99	4.52	5.01E-05	4.23E-03
UBERON:0000204	*Ventral part of telencephalon*	102	25	10.66	2.35	3.48E-05	3.36E-03
UBERON:0002946	*Regional part of cerebellum*	79	19	8.26	2.30	3.86E-04	2.74E-02
UBERON:0002757	*Regional part of epithalamus*	546	121	57.06	2.12	1.30E-16	8.78E-14
UBERON:0008904	*Neuromast*	169	37	17.66	2.10	8.91E-06	1.09E-03
UBERON:0000203	*Pallium*	105	23	10.97	2.10	4.27E-04	2.88E-02
UBERON:0003895	Hypaxial myotome region	101	22	10.55	2.09	6.13E-04	3.76E-02
UBERON:0010134	*Secretory circumventricular organ*	478	104	49.95	2.08	8.10E-14	3.64E-11
UBERON:0003902	*Retinal neural layer*	633	136	66.15	2.06	2.09E-17	2.82E-14
UBERON:0003296	*Gland of diencephalon*	559	115	58.42	1.97	1.70E-13	5.73E-11
UBERON:0002540	*Lateral line system*	519	101	54.24	1.86	6.78E-06	9.15E-04
UBERON:0001898	*Hypothalamus*	329	59	34.38	1.72	6.10E-04	3.76E-02
UBERON:0000045	*Ganglion*	596	105	62.28	1.69	2.26E-08	5.07E-06
UBERON:0002199	Integument	565	97	59.04	1.64	3.51E-07	5.92E-05
UBERON:0001894	*Diencephalon*	1,459	239	152.47	1.57	1.82E-03	9.82E-02
UBERON:0005725	*Olfactory system*	760	124	79.42	1.56	6.94E-08	1.34E-05
UBERON:0002298	*Brainstem*	624	101	65.21	1.55	3.79E-05	3.41E-03
UBERON:0003051	Ear vesicle	843	133	88.09	1.51	9.98E-07	1.50E-04
UBERON:0000489	Cavitated compound organ	1,701	255	177.76	1.43	1.34E-08	3.63E-06
UBERON:0002028	*Hindbrain*	1,600	225	167.2	1.35	7.71E-05	6.11E-03
UBERON:0000479	Tissue	4,751	645	496.49	1.30	1.29E-03	7.27E-02
UBERON:0000955	*Brain*	3,255	435	340.15	1.28	1.29E-04	9.70E-03
UBERON:0000483	Epithelium	3,734	489	390.21	1.25	8.56E-04	5.02E-02

Note.—Anatomical structures are sorted by their enrichment fold compared with null expectation. The substructures of the “broad” nervous system are highlighted in italics. The “weight” algorithm of the topGO package was used to decorrelate the structure of the ontology. The full list of anatomical structures, sorted by *P*-value, is shown in supplementary table S1, Supplementary Material online.

A high fraction of the enriched anatomical structures were neural (e.g., subparts of the telencephalon, cerebellum, epithalamus, neuromast, retinal neural layer). To test whether this did not simply reflect the structure of the Uberon anatomical ontology, which could use more terms to describe the nervous system than other anatomical systems, we made the inventory of all nervous system structures described in the ontology. We built two lists, a strictly defined list, and a broader one also including sensory systems as well as embryonic precursors of nervous structures (“strict” and “broad” lists; see Materials and Methods). Using these reference lists we observed that among the 25 structures shown to be significantly enriched for the expression of 3R ohnologs, 19 were part of the nervous system (broad list; of which 15 were part of the strict list). These proportions were significantly higher than the proportion of nervous system structures among all tested structures (Fisher’s exact tests; broad list: *P *=* *0.0001, with odds-ratio = 5.41; strict list: *P *=* *3.2e-5, odds-ratio = 5.67). Even among structures that were not part of the nervous system, some still shared the same ectoderm developmental origin as nervous system (e.g., “ear vesicle” or “integument”).

We applied the same procedure to other anatomical systems (see Materials and Methods), but they were always underrepresented among the structures enriched for the expression of 3R ohnologs. We also verified that the overrepresentation of nervous system structures was not dependent on the FDR threshold used in the enrichment analysis. In the rest of the article we describe the ontology enrichment results obtained at a FDR threshold of 10%, and we use the broad list of nervous system structures as reference.

We next turned to singleton genes, whose duplicate copy was lost after the 3R whole-genome duplication, and investigated if these were preferentially expressed in any anatomical structure. We found only two structures enriched for this group of genes: “unspecified” and “alar plate midbrain.” However, 35 structures were significantly depleted in expression of singletons (supplementary table S2, Supplementary Material online), of which 22 were part of the nervous system (Fisher’s exact test; *P *=* *0.0024, odds = 2.89)

In summary, we observed that genes retained in duplicate after the fish-specific (“3R”) whole-genome duplication were strongly biased for nervous system expression (in very diverse structures, including developmental precursors and sensory organs), whereas genes that were not retained in duplicate had the opposite tendency to not show expression in these structures. We reproduced these analyses using an independent data set of 3,212 and 10,415 zebrafish 3R ohnologs and singletons identified using phylogenetic and synteny analyses (Braasch etal. 2016), and obtained consistent results (supplementary tables S3 and S4, Supplementary Material online).

### Pre or Postduplication Bias?

These results could be explained by a duplicate retention bias, that is, genes expressed in the nervous system before 3R were more likely retained as ohnologs. Or they could be explained by a bias in postduplication evolution, that is, ohnologs were more likely to acquire expression in the nervous system. To disentangle these two scenarios, it is possible to focus on an outgroup species that did not experience the whole-genome duplication, and compare the properties of orthologs of ohnologs to orthologs of singletons in this species, as a proxy for the preduplication properties of zebrafish genes (Davis and Petrov 2004; Brunet etal. 2006; Roux and Robinson-Rechavi 2008). The mouse represents a convenient such outgroup, since a large number of in situ hybridization data are also available for this species, allowing to test the enrichment of expression in anatomical structures using the same methodology (see Materials and Methods).

We found that mouse orthologs of zebrafish 3R ohnologs were enriched for expression in 57 anatomical structures, among which 46 were nervous system structures (supplementary table S5, Supplementary Material online; *P *=* *1.6e-19, odds = 13.9). In parallel, mouse orthologs of 3R singletons were significantly depleted for expression in two nervous structures, “olfactory cortex mantle layer” and “CA2 field of hippocampus,” and just above significance threshold, nervous system structures were also almost exclusively present at the top of the list (supplementary table S6, Supplementary Material online).

These results, consistent with the observations in zebrafish, suggest that the nervous system enrichment can be explained by an ohnolog retention bias, and that expression patterns before the 3R whole-genome duplication, or in an outgroup, can predict this retention bias.

### The Nervous System Bias Is Weakly Detected for 2R Ohnologs

We repeated the enrichment analysis with mouse 2R ohnologs identified by phylogenomics, but these genes did not show any significant enrichment (supplementary table S7, Supplementary Material online). However, there was a significant enrichment for nervous system structures when we used an independent list of 5,376 mouse 2R ohnologs (Singh etal. 2015), identified using synteny comparison across multiple genomes (supplementary table S8, Supplementary Material online; 91 nervous structures out of 297 enriched structures; *P *=* *0.0081, odds = 1.44).

Mouse 2R singletons were depleted in 107 structures, 31 of which belonged to the nervous system (supplementary table S9, Supplementary Material online). This slight overrepresentation of nervous system structures was however not significant (*P *=* *0.25, odds = 1.29).

In summary, the results from the 2R whole-genome duplications were consistent with those from the 3R whole-genome duplication. Several technical factors could account for the fact that the 2R trends were weaker than the 3R trends, notably the older age of these events, but also postduplication evolution patterns confounding this analysis, that was not performed in an outgroup species.

### Small-Scale Duplications

We also investigated whether an anatomical expression bias existed for duplicate genes that arose from other sources than whole-genome duplications, that is, small-scale duplications. Because there was no whole-genome duplication in the phylogenetic branches leading to the zebrafish and mouse lineages, after 3R and 2R, respectively, we isolated duplicates dated to these branches as small-scale duplicates. We removed those that were specific to these species since they could still be polymorphic or represent errors in the genome assemblies (see Materials and Methods). Unfortunately, the small number of genes identified (385 and 646 duplicate genes for zebrafish and mouse, respectively) led to low power of the enrichment test. In both species, we did not detect any significantly enriched or depleted anatomical structure. In mouse, the depletion results were close to significance, and we noticed numerous nervous system structures present at the top of the list (supplementary table S10, Supplementary Material online). Using an external curated list of small-scale duplicate pairs specific to rodents (Farre and Alba 2010), there were four structures with a significant expression enrichment (“placenta,” “stomach glandular region mucosa,” “ectoplacental cone,” and “cardia of stomach”) and eight structures with a significant depletion, seven of which were part of the nervous system (*P *=* *0.0003, odds = 22.1; supplementary table S11, Supplementary Material online). Overall, there was weak evidence for a nervous system expression bias of small-scale duplicates.

### Validation with Microarray Data

**Fig. 1.**
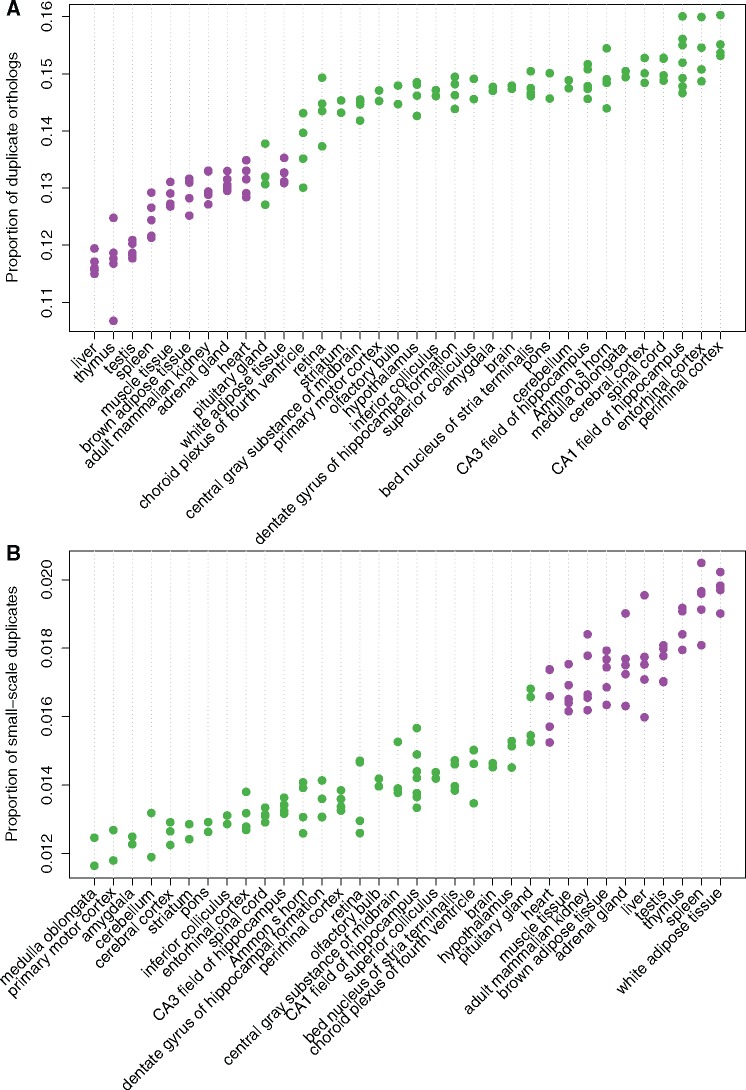
Proportion of mouse orthologs of zebrafish 3R ohnologs among genes expressed in the different tissues sampled in the GSE3594 microarray experiment. The reference gene set was composed only of mouse orthologs of zebrafish 3R ohnologs and singletons. Tissues are ranked based on the average proportion of orthologs of ohnologs expressed. Each dot represents a sample (biological replicate). Green color represents nervous-system tissues and purple represents nonnervous-system tissues.

To check whether the expression biases could be observed with other types of expression data, we retrieved a microarray data set in mouse that included samples from multiple nervous and nonnervous tissues (see Materials and Methods). We called the genes expressed or not in each sample, and ranked the tissues based on the proportion of mouse orthologs of zebrafish 3R ohnologs expressed (fig. 1*A*). We observed that the samples expressing the highest proportion of orthologs of ohnologs belonged to the nervous system. This result was confirmed with another microarray data set (supplementary fig. S2*A*, Supplementary Material online), and with human RNA-seq data from the GTEx consortium (supplementary fig. S2*B*, Supplementary Material online).

Across neural tissues, there was little variation in the proportion of orthologs of ohnologs expressed, confirming that this bias was general to the whole nervous system. Only a few neural tissues stood out with lower proportions, notably the pituitary gland (fig, 1*A*, supplementary figs. S2*A* and S2*B*, Supplementary Material online). An independent microarray data set, including samples from 46 neural tissues confirmed that the pituitary gland, but also the pineal body, displayed a lower proportion of orthologs of ohnologs expressed (supplementary fig. S2*C*, Supplementary Material online). Interestingly, these tissues also stood out from clustering analyses based on expression levels across numerous nervous tissues (Zapala etal. 2005; Kasukawa etal. 2011), possibly because of their secretory activities (Gu and Su 2007) or different cell type composition.

Finally we ranked tissues based on the proportion of small-scale duplicates expressed, and observed an opposite picture: tissues expressing the lowest proportion of small-scale duplicates belonged to the nervous system (fig. 1*B*), supporting the weak trend observed with in situ hybridization data.

### The Rate of Protein Sequence Evolution Is Associated with Ohnolog Retention

The nervous system expression bias could be an indirect effect of other factors driving differential retention of duplicate genes. For example, it was observed that genes with slow rates of amino acid substitution were more retained as ohnologs (Davis and Petrov 2004; Brunet etal. 2006). Since genes expressed in the nervous system also tend to be slowly evolving (Duret and Mouchiroud 2000; Gu and Su 2007; Drummond and Wilke 2008; Kryuchkova-Mostacci and Robinson-Rechavi 2015), the rate of amino acid substitutions could be a confounding factor behind the expression bias.

**Fig. 2.**
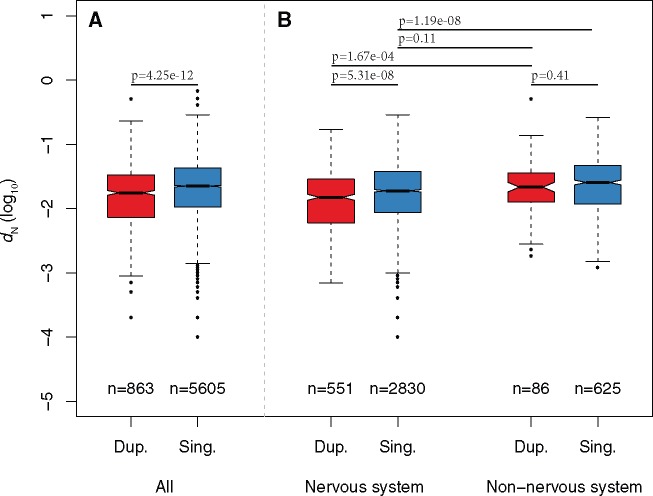
(*A*) Comparison of the rate of protein sequence evolution (*d*_N_, plotted in log_10_ scale) for mouse orthologs of zebrafish 3R ohnologs (“Dup.”) or singletons (“Sing.”). The number of genes in each category is indicated below each box. The *P*-values from a Wilcoxon test comparing categories are reported above boxes. The lower and upper intervals indicated by the dashed lines (“whiskers”) represent 1.5 times the interquartile range, or the maximum (respectively minimum) if no points are beyond 1.5 IQR (default behavior of R function boxplot). (*B*) Similar to (*A*), but mouse orthologs of zebrafish 3R ohnologs and singletons are split according to their expression in the nervous system (“Nervous system” and “nonnervous system”). The numbers of duplicates and singletons genes do not add up to numbers of genes in (*A*) because only genes with in situ hybridization data were used for this analysis.

**Fig. 3.**
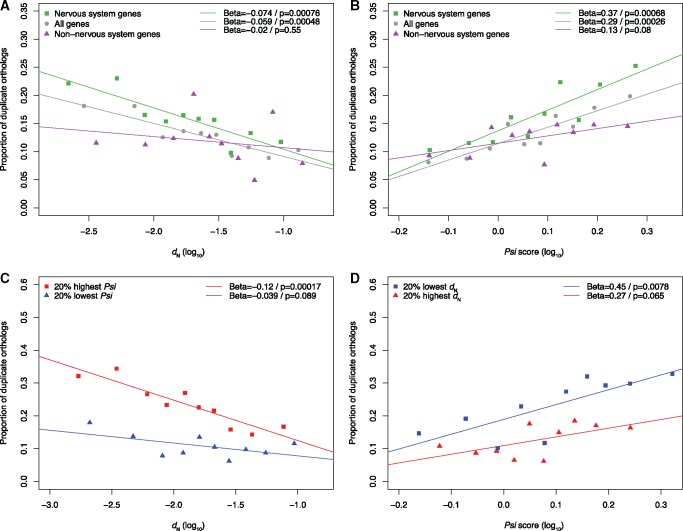
(*A*) Relation between proportion of mouse orthologs of zebrafish 3R ohnologs and rate of nonsynonymous substitution. Genes were split into ten equal-sized bins of *d*_N_, and the median *d*_N_ of each bin was plotted on the *x*-axis (in log_10_ scale). A linear regression was fit to the ten data points, whose slope (Beta value) and *P*-value are indicated in the top-right corner of the plot. The analysis including all genes is plotted in grey and circles, whereas the analysis including only nervous system genes is plotted in green and squares and the analysis including only nonnervous system genes is plotted in purple and triangles. (*B*) Relation between proportion of mouse orthologs of zebrafish 3R ohnologs and Akashi’s test *Psi* score, for nervous and nonnervous system genes. Legend similar to (*A*). (*C*) Similar to (*A*), using only nervous system genes, divided in two groups: the 20% genes with lowest *Psi* score plotted in blue and squares, and the 20% genes with highest *Psi* score plotted in red and triangles. (*D*) Similar to (*B*), using only nervous system genes, divided in two groups: the 20% genes with lowest *d*_N_ plotted in blue and squares, and the 20% genes with highest *d*_N_ plotted in red and triangles.

We first verified using our data set that the 10% genes with the lowest nonsynonymous substitutions rate values (*d*_N_, calculated from pairwise comparisons of mouse-rat orthologs) were indeed significantly enriched for expression in nervous structures (supplementary table S12, Supplementary Material online; *P *=* *8.9e-7, odds = 2.13). We also verified that mouse orthologs of zebrafish 3R ohnologs had a lower *d*_N_ than orthologs of singletons (fig. 2*A*). We then subdivided the orthologs based on their expression in the nervous system, and surprisingly, while the pattern of slower rate of evolution of orthologs of ohnologs held among nervous system genes, it did not among nonnervous system genes (fig. 2*B*). This result was confirmed when we split nervous system and nonnervous system genes into ten equal-sized bins of *d*_N_ (i.e., bins with equal numbers of genes): the proportion of orthologs of ohnologs in each bin was significantly associated with *d*_N_ for nervous system genes, but not so for nonnervous system genes (fig. 3*A*).

Since there are more than four times the number of nervous system genes than nonnervous system genes in our analysis, we verified that this results could not be explained by power issues (supplementary text, Supplementary Material online). We also verified that this pattern held when limiting the set of nervous-system genes to those that were not expressed in any nonnervous structure (supplementary fig. S3, Supplementary Material online).

In summary, the biological signal of differences of sequence evolution rates between ohnologs and singletons, apparent at the whole-genome level in our analysis and previously reported in other studies, is in fact mainly caused by nervous system genes. These greatly outnumber nonnervous system genes, for which no sequence evolution rate difference is observed between ohnologs and singletons.

### Highly Expressed Nervous System Genes Are More Retained as Ohnologs

The main hypothesis to explain the slow rates of sequence evolution of nervous-system genes is that their protein sequence was optimized over the course of evolution (Drummond etal. 2005; Drummond and Wilke 2008; Zhang and Yang 2015; Wang 2016). Preferred amino-acids minimize the levels of misfolded proteins, which are toxic to cells because they are prone to aggregate to other proteins and to hydrophobic surfaces such as membranes (Drummond and Wilke 2008; Yang etal. 2012). Preferred amino-acids also minimize the levels of proteins misinteracting with other proteins (Yang etal. 2010).

The long lifetimes and high membrane surface area of neurons make them particularly vulnerable to damages of toxic proteins, explaining why in vertebrates the amino-acid sequences of nervous system genes are the most optimized and conserved (Drummond and Wilke 2008, 2009; Biswas etal. 2016). We verified using our data set that the negative correlation between *d*_N_ and expression levels was indeed markedly stronger in nervous tissues compared with other tissues (supplementary fig. S4*A*, Supplementary Material online; Duret and Mouchiroud 2000; Drummond and Wilke 2008; Kryuchkova-Mostacci and Robinson-Rechavi 2015).

We then tested whether the association between *d*_N_ and retention rates could be driven by the expression level of genes in the nervous system. To summarize the expression of genes across nervous system tissues, we considered their mean or maximum expression level across the 102 samples from 46 nervous tissues from the GSE16496 microarray experiment (supplementary figs. S4*B*, S5*A*, and S5*B*, Supplementary Material online). We then split genes into ten equal-sized bins of mean and maximum nervous system expression level. The proportion of mouse orthologs of zebrafish 3R ohnologs in the bins was significantly associated with maximum, but not with mean nervous system expression (supplementary fig. S6*A*, Supplementary Material online). Interestingly, the association with maximum nervous expression was maintained when we controlled for *d*_N_ by repeating the same analysis on genes with the highest or lowest *d*_N_ (supplementary fig. S6*B*, Supplementary Material online; although the trend for low *d*_N_ genes was slightly below the significance threshold). Conversely the association between *d*_N_ and retention rate disappeared when we controlled for maximum nervous expression by repeating the *d*_N_ analysis on genes with the highest or lowest maximum nervous expression level (supplementary fig. S6*C*, Supplementary Material online). A small residual *d*_N_ trend was visible: if maximum nervous expression perfectly controlled for *d*_N_, the two regression lines would be overlapping in supplementary figure S6*B*, Supplementary Material online, and they would be flat in supplementary figure S6*C*, Supplementary Material online.

In summary, for nervous system genes, the maximum level of expression in nervous tissues is clearly associated with higher rates of ohnolog retention. It is difficult though from these results alone to assess whether this association is direct or indirectly caused by the association of both factors to *d*_N_.

### Selective Constraints at Synonymous Sites Also Influence Ohnolog Retention

The association of retention rates with *d*_N_, and with maximum nervous expression, suggests a potential link with selection for translational accuracy. In addition to selection for optimal amino-acid sequences of genes, which reduces the levels of toxicity of protein products, synonymous codon usage was also shown to be optimized to increase translational robustness and reduce the levels of mistranslation-induced protein products toxicity (Drummond and Wilke 2008; Yang etal. 2010). Codons binding their cognate tRNA with higher affinity than noncognate competitors are translated more accurately, decreasing the chances of incorporation of wrong amino acids during translation. The selection to maintain a state of optimized synonymous codon usage is apparent in the association between *d*_N_ and *d*_S_ (supplementary fig. S5*C*, Supplementary Material online), and in the stronger negative correlation between the rate of synonymous substitutions (*d*_S_) and expression levels in nervous tissues compared with other tissues (supplementary fig. S7*A*, Supplementary Material online). However, we could not observe any relation between the proportion of mouse orthologs of zebrafish 3R ohnologs and *d*_S_ (supplementary fig. S7*B*, Supplementary Material online).

This may be explained by the fact that selection for translational robustness does not act on synonymous codon usage at all sites, but predominantly on those that are the most important for the structure of the protein (Drummond and Wilke 2008; Zhou etal. 2009) or that are aggregation-prone (Lee etal. 2010). The strength of this effect can be quantified using Akashi’s test, assessing how strong is the association between preferred codons and conserved amino acids, taken as a proxy of constrained sites in the protein (Akashi 1994).

The odds ratio reflecting this association (“*Psi*” score) was only weakly correlated with both the rate of nonsynonymous substitutions, *d*_N_, and with maximum nervous expression (supplementary fig. S5*E* and *F*, Supplementary Material online), but was more strongly associated with translation rates, calculated from ribosome profiling data in embryonic stem cells, embryonic fibroblasts or neutrophils (Dana and Tuller 2014; see Materials and Methods; supplementary fig. S8, Supplementary Material online). Genes showing the 10% highest *Psi* score were enriched for expression in the nervous system (*P *=* *0.00081, odds ratio = 1.98), and interestingly the top structures were almost exclusively developing ectodermal or neural structures (e.g., “rhombomere,” “presumptive midbrain,” “limb bud”; supplementary table S13, Supplementary Material online). In summary, the *Psi* score captures some aspect of selection for translational accuracy that seems largely independent of the constraints on amino acid sequences.

Interestingly, when we separated mouse genes in ten equal-size bins of *Psi*, we observed a significant relation with the proportion of orthologs of ohnologs (fig. 3*B*). And similarly to the *d*_N_ trend, the association was supported for nervous system genes, but not for nonnervous system genes. Focusing on the nervous system genes, we checked whether the *d*_N_ and *Psi* trends were dependent (fig. 3*C* and *D*). The proportion of orthologs of ohnologs was the highest for the genes with the lowest *d*_N_ and the highest *Psi*, suggesting a positive interaction between the effects of *d*_N_ and *Psi*. Nervous system genes that had either high *d*_N_ or low *Psi* included around 10% of orthologs of ohnologs, similarly to nonnervous system genes, but nervous system genes that had both low *d*_N_ and high *Psi* included >30%.

In summary, genes with the most optimized sequences, both at nonsynonymous and at synonymous sites, were more retained in duplicate after whole-genome duplication.

### The Nervous System Bias Is Independent from the Dosage-Balance Hypothesis

The dosage-balance hypothesis was previously proposed to explain ohnolog retention after whole-genome duplication (Freeling and Thomas 2006; Makino and McLysaght 2010; Birchler and Veitia 2012; Singh etal. 2012). Groups of interacting genes, sensitive to relative dosage changes (e.g., members of a protein complex, or genes belonging to the same metabolic pathway) could be maintained in duplicate because the loss of any gene of the group would lead to dosage imbalance and be detrimental (Birchler and Newton 1981; Birchler etal. 2005). Notably, dosage imbalance is expected to impact the formation of protein complexes involving at least two different genes, and composed of at least three subunits (Veitia etal. 2008), so genes involved in such complexes should be more retained after whole-genome duplication.

We tested this using protein complex data from the UniProtKB/Swiss-Prot database (The UniProt Consortium 2015), where complex type and number of subunits are precisely annotated. We split genes into six groups: genes involved in 1) monomers, 2) homo-multimers, and 3) hetero-dimers, which should not be sensitive to dosage imbalance, 4) genes involved in hetero-multimers with at least three subunits, which should be sensitive to dosage imbalance; 5) genes involved in uncharacterized complexes, which likely include some genes sensitive to dosage imbalance; and finally 6) nonannotated genes. Genes annotated to several groups were kept in the group expected to be most sensitive to dosage imbalance (see Materials and Methods). We observed that the proportion of mouse orthologs of zebrafish 3R ohnologs was the highest for members of hetero-multimer complexes, consistent with the dosage-balance hypothesis (supplementary fig. S9*A*, Supplementary Material online). This effect was independent from nervous system expression, since it was observed both for nervous and nonnervous system genes. When controlling for *d*_N_, we observed a positive interaction between the two effects: members of hetero-multimer complexes that had low *d*_N_ were the most retained (supplementary fig. S9*B*, Supplementary Material online).

Another manifestation of the dosage-balance hypothesis could be selection to maintain stoichiometry within metabolic pathways. Previous studies in *Paramecium* and *Arabidopsis* have reported that the retention rate of genes involved in metabolic pathways differed across timescales, and was higher than other genes for recent whole-genome duplication events, whereas it was lower for ancient events (Aury etal. 2006; Gout etal. 2009; Bekaert etal. 2011). Consistent with these observations, we observed a lower proportion of mouse orthologs of zebrafish 3R ohnologs among genes involved in metabolic processes (supplementary fig. S10, Supplementary Material online), both for nervous and nonnervous system genes, confirming that there is no long-term action of selection against dosage imbalance on whole pathways.

Finally, we examined the relation between the level of protein connectivity and retention rates. The number of protein–protein interactions was previously taken as a proxy for sensitivity to dosage imbalance (Prachumwat and Li 2006; Flagel and Wendel 2009; Rodgers-Melnick etal. 2013; Cuypers and Hogeweg 2014). We observed that mouse orthologs of zebrafish 3R ohnologs had a significantly higher connectivity than orthologs of singletons (supplementary fig. S11*A*, Supplementary Material online), in agreement with previous studies (Hakes etal. 2007; Liang and Li 2007; Rodgers-Melnick etal. 2012). But similarly to the *d*_N_ trend, when we subdivided genes based on their expression pattern, the trend held only for nervous system genes. Since highly connected genes tend to display a lower *d*_N_ (supplementary fig. S5*G*, Supplementary Material online), we tested whether the relation between retention rate and connectivity could be explained by *d*_N_ differences: this was not the case, with connectivity and *d*_N_ even positively interacting to explain retention rates (supplementary fig. S11*B* and *C*, Supplementary Material online). Similarly, the connectivity trend could not be explained by other weakly correlated factors, maximum nervous expression and *Psi* score (supplementary fig. S5*I* and *H*, Supplementary Material online), but it disappeared when we split genes based on their annotation the six complex subtypes (supplementary fig. S11*D*, Supplementary Material online).

This suggests that the connectivity trend could indeed be explained by higher dosage sensitivity of most highly connected genes, although there is no clear a priori reason for the trend to be seen only among nervous system genes (see Discussion).

### Small-Scale Duplication Is Not Associated to the Same Underlying Factors

Small-scale duplicates have often been observed to behave in an opposite way to ohnologs, a pattern that we confirmed with the lower rate of duplication of genes expressed in the nervous system (fig. 1*B*). More careful examination indicated that this bias was not caused by the same underlying factors as ohnologs.

First, there was a difference in *d*_N_ values between genes that experienced a small-scale duplication event and other genes, but this was true both for nervous system and nonnervous system genes (supplementary fig. S12*A*, Supplementary Material online). This suggests that the depletion for nervous system expression might just be an indirect consequence of the association between small-scale duplication and sequence evolution rates. Second, the relation between the proportion of small-scale duplicates and *d*_N_ was best explained by a linear fit, whereas the best model was a log-linear trend for ohnologs (supplementary fig. S12*B*, Supplementary Material online). Third, there was no relation between the proportion of small-scale duplicates and *Psi* score, suggesting that there was no association between selection for translational accuracy and small-scale duplication patterns (supplementary fig. S12*B* and *C*, Supplementary Material online).

Since expression level is a major determinant of *d*_N_, we examined the relation between the proportion of small-scale duplicates and summaries of expression levels across nervous and nonnervous tissues. The best trend was obtained using the average expression level across all available tissues (not only nervous tissues; supplementary fig. S13*A*, Supplementary Material online). There was even a positive interaction between the effects of *d*_N_ and average expression level: genes with a small-scale duplication history displayed both a low average expression and a high *d*_N_ (supplementary fig. S13*B* and *C*, Supplementary Material online).

We finally tested the dosage-balance hypothesis on small-scale duplicates, using protein complex information. In comparison to whole-genome duplication patterns (supplementary fig. S9, Supplementary Material online), hetero-multimer genes displayed a lower proportion of duplicates, consistent with their dosage-sensitivity (supplementary fig. S14, Supplementary Material online), but this was only true for nervous system genes. For the subset of nonnervous system genes, the proportion of duplicate hetero-multimer genes was surprisingly higher than the other groups of genes. Given that this category includes the lowest number of genes, this pattern must be interpreted carefully. The low number of small-scale duplicates makes it unfortunately difficult to reliably test the dependency of this trend with respect to the *d*_N_ and average expression level trends.

## Discussion

In this study we took advantage of thousands of high quality in situ hybridization data describing precisely mouse and zebrafish gene expression patterns. These are mapped to ontologies describing the anatomy of these species, making it possible to perform ontology enrichment tests and to detect tissues enriched for the expression of genes of interest. This methodology corrects for biases in annotation and in data availability, that is, some anatomical structures are better annotated than others (Yon Rhee etal. 2008).

We uncover a strong and robust trend whereby genes expressed in neural tissues are more likely retained in duplicate after whole-genome duplication. These same genes are less likely to duplicate via small-scale duplication events. To our knowledge, this result was never previously reported, but is fully consistent with previous studies. For example, ohnologs were found enriched for Gene Ontology terms related to signaling, behavior, neural activity, or neurodevelopment (Brunet etal. 2006; Putnam etal. 2008; Roux and Robinson-Rechavi 2008; Kassahn etal. 2009; Huminiecki and Heldin 2010; Howe etal. 2013; Schartl etal. 2013), which are typical nervous system genes functions. The slow rate of sequence evolution of ohnologs (Davis and Petrov 2004) can also be explained by the tendency of nervous system genes to evolve slowly (Duret and Mouchiroud 2000; Gu and Su 2007; Drummond and Wilke 2008).

Surprisingly, there have been few previous analyses of ohnolog retention biases with respect to gene expression patterns, probably because of the limited anatomical resolution of most microarray and RNA-seq data sets, and the difficulty in gathering many in situ hybridization experiments for an integrated analysis. Satake etal. (2012) reported that the proportion of 2R ohnologs detected in EST data sets was the highest in ectoderm-derived tissues, whereas the proportion of small-scale duplicates was the lowest, which is consistent with our observations.

Once this pattern was established, the next challenging task was to disentangle, within the network of factors associated with retention rates, which factors could be causal, and more broadly, which mechanisms are in action (Drummond etal. 2006; Singh etal. 2012; Kryuchkova-Mostacci and Robinson-Rechavi 2015). These analyses were done in mouse, an outgroup species used as proxy for the preduplication state in the teleost fish ancestor. Unfortunately we lack at present good data to verify these patterns in teleosts, that is, we lack closely related genomes to zebrafish, or at least fish genomes outgroup to the 3R whole-genome duplication with sufficient functional data.

The rate of nonsynonymous substitutions, *d*_N_, is strongly associated to the maximum level of expression across nervous tissues, an association that is likely caused by selection for optimized amino-acid sequences against the toxic effects of misfolded or misinteracting protein products (Drummond and Wilke 2008; Zhang and Yang 2015). Interestingly, both factors are independently associated with retention of nervous system ohnologs, suggesting that selection for optimized amino-acid sequences could play a key role in this process. This hypothesis is corroborated by the observation that another manifestation of selection against toxic protein products, the optimization of codon usage at structurally sensitive sites to increase translational robustness, is also associated with retention rates, and this effect is not controlled by *d*_N_ or maximum nervous system expression. All these effects even seem to be positively interacting: genes that have a low *d*_N_, a high maximum nervous system expression and a high *Psi* score have the highest chances of retention.

**Fig. 4.**
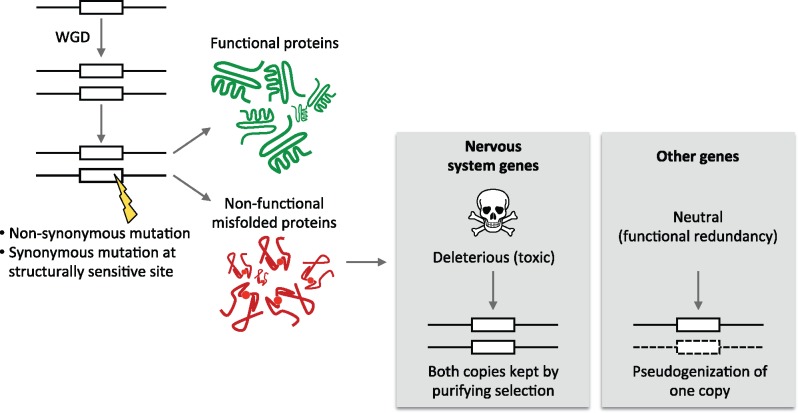
Illustration of the model proposed to explain favored retention of nervous system genes after whole-genome duplication. Nonsynonymous mutations or synonymous mutations at structurally sensitive sites on one duplicate copy can cause an increase in the production of nonfunctional toxic protein products. This is likely neutral in most tissues, since the function loss is backed-up by the other copy. This could however be deleterious for nervous system genes because they are expressed in nonrenewing cells, sensitive to the toxic effects of misfolded or misinteracting proteins. Purifying selection will thus prevent the fixation of such mutations, and indirectly contribute to the preservation of both ohnologs.

After whole-genome duplication, duplicate gene loss starts with the fixation of loss-of-function mutations in one of the gene copies. This can occur neutrally as long as the gene function is backed-up by the other copy. Thereafter, the nonfunctional copy accumulates other substitutions and degenerates (Albalat and Canestro 2016). Such a neutral scenario might not be possible for nervous system genes whose sequence is constrained by selection. Indeed, mutations occurring both at nonsynonymous sites, and at some synonymous sites, can increase the rate of production of toxic proteins, and this deleterious effect should hamper their fixation in the population (fig. 4). This simple model can explain how both duplicate gene copies can be “protected” from degeneration by purifying selection after whole-genome duplication, despite functional redundancy.

More broadly than nervous system genes, any gene subject to dominant deleterious effects mutations should be more likely retained as ohnolog, since the organism would pass by a low fitness intermediate when losing one copy. In fact, Gibson and Spring (1998), and later Singh etal. (2012) proposed such a model to explain the puzzling observation that disease-causing genes were preferentially retained after the 2R whole-genome duplications (Gibson and Spring 1998; Makino and McLysaght 2010; Dickerson and Robertson 2012; Singh etal. 2012, 2014; Chen etal. 2013; Malaguti etal. 2014; Tinti etal. 2014). They later supported this model by theoretical population genetics work (Malaguti etal. 2014), explaining the accumulation of repertoires of “dangerous” genes after whole-genome duplication. This is also supported by the enrichment of ohnologs among genes for which copy number variants are pathogenic (Rice and McLysaght 2017).

Of course, our model does not totally exclude the possibility of pseudogenization of nervous system genes. For example, a mutation introducing a stop codon at the very beginning of the coding sequence is not likely to produce toxic products. It is also possible that regulatory mutations first silence one duplicate copy, opening the way to its neutral degeneration (Thompson etal. 2016). The neutral evolution of asymmetric expression levels between duplicate copies has indeed been reported (Gout and Lynch 2015; Lan and Pritchard 2016; Thompson etal. 2016). But (1) this process was shown to require substantial amounts of time, and (2) the evolution of expression levels in the nervous system is tightly controlled and slower than in other tissues (Pennacchio etal. 2006; Brawand etal. 2011; Barbosa-Morais etal. 2012; Merkin etal. 2012). Hence, there is little reason to think that other pseudogenization routes would compensate the deficit of losses for nervous system ohnologs.

Our model also does not exclude the possibility that some ohnolog pairs are retained through the action of previously described mechanisms (Innan and Kondrashov 2010), for example subfunctionalization (Force etal. 1999) or neofunctionalization (Ohno 1970; He and Zhang 2005). We could not find any reasonable explanation for the nervous system retention bias using these alternative mechanisms, but these might however be necessary to maintain ohnologs in the long term. For example, nervous system duplicates that avoided rapid initial loss could be eventually retained because they evolved new functions later in time (Kassahn etal. 2009; Chen etal. 2011).

Another interesting model is the dosage-balance hypothesis, which was proposed to be a major determinant of duplicate gene retention after whole-genome duplication, at least on short evolutionary time scales (Papp etal. 2003; Freeling and Thomas 2006; Makino and McLysaght 2010; McLysaght etal. 2014; Thompson etal. 2016). This hypothesis is difficult to test in vertebrates because there are only a few noisy data sets allowing to assess the sensitivity of genes to dosage imbalance. Previous studies have sometimes relied on indirect evidence; for example, it was found that genes with high levels of protein–protein interactions (more connected genes) tended to be more retained after whole-genome duplication, which was interpreted as an evidence that these genes are more sensitive to changes in dosage (Liang and Li 2007; Rodgers-Melnick etal. 2013).

Such an interpretation is subject to caution. Although we indeed found connectivity to be significantly associated with retention rates, we noticed that the trend was only supported for nervous system genes. There is a priori no reason to expect this pattern from the dosage-balance hypothesis. However, it was shown that amino-acid sequences are optimized to reduce the levels of misinteraction with other proteins (Yang etal. 2012), an effect that might be more important for highly connected proteins, and for those expressed in nonrenewing neural cells than other cell types. Protein surface residues in particular are optimized for decreased stickiness and misinteractions, which are deleterious because they waste functional molecules, can interfere with functional interactions, or initiate damaging cellular processes (Zhang etal. 2008; Vavouri etal. 2009; Yang etal. 2012). The chances of detrimental effect might be higher for highly connected proteins, and similarly to protein misfolding, the effects might be more detrimental to nonrenewing neural cells than other cell types, contributing to a retention bias of highly connected nervous system ohnologs. Hence, this mechanism provides an alternative explanation, probably complementary to the dosage-balance hypothesis, to the relation between connectivity and retention rates.

A better source of evidence to test the dosage-balance hypothesis is protein complex data. But different complex subtypes are not equally sensitive to dosage imbalance (Veitia etal. 2008). When separating complexes into permanent or transient complexes, a previous study in human surprisingly observed that the retention rates after the 2R whole-genome duplications were lower for permanent complexes, despite their higher susceptibility to dosage-balance constraints (Singh etal. 2012). We separated genes into those involved or not in dosage-sensitive complexes and, consistent with the dosage-balance hypothesis, observed a higher retention of the former. Moreover, this trend was supported both for nervous system and nonnervous system genes, and was independent of confounding factors such as *d*_N_, suggesting that the effects of selection against gene dosage imbalance on ohnologs retention are likely independent from the effects of selection against toxic protein products.

Another analysis that we performed with another source of data gave somewhat conflicting results, but the annotations were less comprehensive and precise (see supplementary text, Supplementary Material online). This underlines that careful analysis and high-quality data sets are needed to study the effects of selection against gene dosage imbalance on ohnologs retention independently from the effects of selection against toxic protein products. For example, it is important to be careful with data transferred across species, which could be biased by the rates of sequence evolution, and to study preduplication biases in an outgroup species, because duplication itself likely influences postduplication evolution of dosage-sensitive genes (e.g., ohnologs might be more likely to evolve into hetero-multimer complexes members; Musso etal. 2007; Qian and Zhang 2014).

Interestingly, it is worth noting that the dosage-balance hypothesis, quite similarly to our model of figure 4, also explains duplicate retention biases by the action of purifying selection (Freeling and Thomas 2006), acting not on detrimental mutations in coding sequences as in our model, but also on detrimental changes in expression of dosage-sensitive genes. The predominant role of purifying selection can account for the observation that ohnologs usually do not duplicate via small-scale duplication events. Indeed, small-scale duplication events first need to reach fixation in the population, a process that is rarely successful for such genes, whose mutations can be dominant negative (Innan and Kondrashov 2010; Singh etal. 2012).

A recent study (Rice and McLysaght 2017) has reported that genes found in pathogenic copy number variant mutations are involved in development, enriched in protein complexes, have high expression, and have evolutionary patterns depleted in small-scale duplications but enriched in ohnologs. These observations are consistent with dosage-balance, and interpreted in that manner (Rice and McLysaght 2017). Yet, interestingly, the “class P” (pathogenic) genes of Rice and McLysaght (2017) have expression highly enriched in nervous system structures by TopAnat (not shown). It is possible that dosage imbalance effects might be more severe in the nervous system than other tissues, but (1) there is to our knowledge no prior report of this effect, and (2) we did not observe an underrepresentation of small-scale duplications in nervous system ohnologs compared with nonnervous system ohnologs (supplementary text, Supplementary Material online). Thus the observations of Rice and McLysaght (2017) could rather be at least in part explained by our hypothesis of selection against the toxicity of protein products.

We observed that small-scale duplicates were rarely expressed in the nervous system, but this time, likely as an indirect effect of low fixation and retention rates of duplicates of slowly evolving highly expressed genes. This is consistent with purifying selection acting primarily on the deleterious effects of doubling the gene expression induced by small-scale duplications (Schuster-Böckler etal. 2010; McLysaght etal. 2014; Rice and McLysaght 2017). Although average expression level is highly correlated with *d*_N_, it did not account for the entirety of the relation between *d*_N_ and rate of small-scale duplication. The additional effect of *d*_N_ could be due to postduplication biases, that we did not control for in this analysis. Small-scale duplicates were indeed shown to experience an accelerated evolutionary rate after duplication, possibly associated with a process of sub or neofunctionalization (Jordan etal. 2004; Fares etal. 2013; Pegueroles etal. 2013). Finally, selection against protein misfolding was not associated with small-scale duplication rates. This is perhaps not surprising, because the sequence of genes expressed in tissues sensitive to protein misfolding was optimized by natural selection, and duplication is unlikely to affect this, especially since the fixation phase of duplicates is probably too short for point mutations to accumulate.

## Conclusion

The implications of our results are manifold. First, they confirm that whole-genome duplication is a unique type of evolutionary event, which enriches the gene set of a lineage with genes under strong purifying selection, for example, dosage-sensitive genes, disease-causing genes, or nervous system genes. Mutations affecting the sequences or the expression of these genes can have clear detrimental consequences, adding a long term burden to genomes. Counter-intuitively, the preferential retention of these genes is driven by the action of purifying selection alone, although this is usually viewed as a protective force. Our study focused on vertebrates, but such a situation is most likely true for other organisms which experienced whole-genome duplications, such as plants or unicellular eukaryotes, although the sets of retained genes might differ.

On the other hand, whole-genome duplications have often been claimed to be beneficial in the long term, since the addition of new genes to genomes provides new material for evolution to act on, and increases evolvability of the lineages (Van de Peer etal. 2009; Kondrashov 2012; Cuypers and Hogeweg 2014). A particularly interesting example is the ancestral 2R event, which added to the genomes of vertebrates a large number of regulatory genes, such as transcription factors, as an indirect effect of purifying selection for gene dosage balance. Freeling and Thomas coined this phenomenon a “spandrel” of purifying selection, and suggested that it contributed to the increased morphological complexity of vertebrates (Gould and Lewontin 1979; Freeling and Thomas 2006).

Our results highlight that another such by-product of purifying selection is the enrichment of the vertebrate genomes for nervous system genes, at a time which coincided with major evolutionary novelties of the nervous system. The expanded toolkit of nervous genes likely provided opportunities for regulatory network rewiring and new functions to evolve (Evlampiev and Isambert 2007; Oakley and Rivera 2008; Chakraborty and Jarvis 2015). For example, it was suggested that the 2R events gave vertebrates the tools to evolve new structures such as the neural crest, placodes, and a midbrain–hindbrain boundary organizer (Holland 2009). Similarly, in fish it was suggested that the 3R whole-genome duplication contributed to expand the toolkit of cognition-related genes that gave teleosts a high level of behavioral complexity compared with other groups of cold-blooded vertebrates such as amphibians and reptiles (Schartl etal. 2013).

## Materials and Methods

Data files and analysis scripts are available on our GitHub repository: https://github.com/julien-roux/Roux_Liu_and_Robinson-Rechavi_2016.

### Mouse and Zebrafish In Situ Hybridization Data

Mouse (*Mus musculus*) RNA in situ hybridization expression data were retrieved from the GXD database (Smith etal. 2007, 2014) in December 2014. Wild-type data, obtained under nonpathological conditions, and with no treatment (“normal” gene expression) were integrated into Bgee (http://bgee.org/), a database allowing the comparison of transcriptome data between species (Bastian etal. 2008). The data used in this article all come from the release 13 of Bgee. In Bgee, expression data are mapped to the Uberon anatomical ontology (http://uberon.org). The mapping from the EMAP (Bard etal. 1998) and MA (Hayamizu etal. 2005) mouse anatomical ontologies (onto which GXD in situ hybridization data are mapped) to Uberon was obtained from Uberon cross-references. Terms from the EMAPA and MA ontologies that were not present in the Uberon ontology, but to which in situ hybridization data were mapped were also included in the analyses.

Similarly, zebrafish (*Danio rerio*) in situ hybridization expression data were retrieved from the ZFIN database (Sprague etal. 2006) in December 2014 and integrated into Bgee release 13 after mapping to the Uberon anatomical ontology. Terms from the ZFA ontology that were not present in the Uberon ontology, but to which in situ hybridization data were mapped were also included in the analyses.

### Mouse Microarray Data

Mouse microarray data and their mapping to the Uberon anatomical ontology were retrieved from Bgee release 13. We targeted experiments including a large number of samples from many different tissues, and including multiple nervous and nonnervous system tissues. We retained the accessions GSE3594, GSE10246, and GSE16496.

GSE3594 is a data set composed of 129 samples from 24 neural tissues and ten body tissues from different strains of inbred mice (Zapala etal. 2005). This experiment was hybridized to the Affymetrix Murine Genome U74A Version 2 array. Raw data (CEL files) were not available from GEO, so the normalized intensities and present/absent calls provided by the MAS5 software (Liu etal. 2002) were used.

GSE10246 corresponds to the GNF Mouse GeneAtlas V3 (Su etal. 2004) and there were 91 samples from 45 tissues (including 12 neural tissues, as well as 7 substructures of the eye) included into Bgee. This data set was hybridized to the Affymetrix Mouse Genome 430 2.0 Array chip and was reprocessed through the Bgee pipeline (see http://bgee.org/bgee/bgee?page=documentation). Briefly, this includes normalization of the signal of the probe sets by the gcRMA algorithm, and a Wilcoxon test on the signal of the probes sets against a subset of weakly expressed probe sets to generate present/absent calls (Schuster etal. 2007).

GSE16496 included expression data 102 samples from 46 regions of the mouse central nervous system (Kasukawa etal. 2011). This data set was hybridized to the Affymetrix Mouse Genome 430 2.0 Array chip and also reprocessed through the Bgee pipeline.

We summarized the expression of genes across nervous system tissues by considering for each gene the mean, median or maximum of log_2_ signal across all samples from the GSE16496 experiment. Results were similar when using nervous tissue samples of the GSE3594 (not shown). Because results were similar using the median or the mean expression across nervous tissues, we only show results using the median.

### Human RNA-seq Data

Human RNA-seq data from the GTEx consortium (Melé etal. 2015; The GTEx Consortium 2015) were retrieved from Bgee release 14 (GTEx processed and annotated data available on ftp.bgee.org; full release planned in February 2017). All samples were manually annotated to the Uberon ontology and only healthy samples were retained, based on metadata annotation (e.g., medical history or cause of death). There were 4,860 retained GTEx libraries, mapped to 75 different Uberon terms. The libraries were reprocessed through the Bgee pipeline to generate present/absent calls for each gene. Briefly, RNA-seq reads from each library were pseudo-aligned with Kallisto (version 0.42.4; Bray etal. 2016) to the annotated human transcripts from Ensembl (release 84). Transcript-level TPM estimates were then summed at the gene level. Reads were also pseudo-mapped to a set of 28,573 intergenic regions, located at least 500 bp away from any genic region, and whose size ranged from 2,000 bp to 20,000 bp. The “background” expression signal observed at these regions was used to set a TPM threshold for each library to determine presence/absence calls. At the threshold the ratio of the proportions of intergenic regions called present to the proportion of coding genes called present was set to 5%.

### Identification of Duplicates and Singletons

Gene families were obtained from the Ensembl database release 79 (Hubbard etal. 2009). We used the Perl API to query the Ensembl Compara Gene trees (Vilella etal. 2009) and scan for gene trees with specific topologies. Notably we stringently selected sets of genes with or without duplications on specific branches of the vertebrate phylogenetic tree. We randomly picked a subset of gene trees to verify that they indeed displayed the expected topologies. Below is a description of the selected topologies, which are illustrated in supplementary figure S1, Supplementary Material online. These are dependent on the set of species integrated into Ensembl release 79, accessible at http://mar2015.archive.ensembl.org/info/about/speciestree.html. All genes lists (file gene_lists.zip) and scripts are available on our GitHub repository.

#### Fish-Specific (3R) Whole-Genome Duplication

We first selected subtrees with a basal speciation node dated at the Neopterygii taxonomical level (supplementary fig. S1*A*–*D*, Supplementary Material Online). These subtrees include a spotted gar (*Lepisosteus oculatus*) outgroup, which did not experience the 3R duplication (Braasch etal. 2016), and teleost fish species, which experienced it. We classified zebrafish genes as confident 3R duplicates if the child node of the root of the subtree was a high confidence (score above 50%) duplication node dated at the Clupeocephala taxonomic level, followed by two speciation nodes dated at the Clupeocephala taxonomic level, each delineating a subtree containing no further duplication or loss on the branches leading to zebrafish (i.e., one zebrafish gene per subtree). We classified zebrafish genes as confident 3R singletons if the child node of the root of the subtree was a speciation node dated at the Clupeocephala taxonomic level, with no further duplication or loss on the branches leading to zebrafish. In total we obtained 2,422 ohnologs, and 8,973 singletons.

Of note, our identification of ohnologs is based on phylogeny alone, and does not use any synteny information. Small-scale duplicates that emerged on the Clupeocephala branch will be wrongly incorporated in the list of 3R ohnologs. Given relatively low rate of retention of duplicates originating from small-scale duplication (Lynch and Conery 2000), we ignored this problem in our analyses.

We classified mouse or human genes as confident orthologs of zebrafish 3R ohnologs if there was a two-to-one orthology relationship to a single mouse/human gene at the Euteleostomi taxonomical level. We classified mouse or human genes as confident orthologs of zebrafish 3R singletons orthologs if there was a one-to-one orthology relationship to a single mouse/human gene at the Euteleostomi taxonomical level. In total we obtained 974 mouse orthologs of 3R ohnologs, 6,373 mouse orthologs of 3R singletons, 976 human orthologs of 3R ohnologs, and 6,358 human orthologs of 3R singletons.

#### Vertebrate (2R) Whole-Genome Duplications

It is still debated whether one or two whole-genome duplication events occurred at the base of vertebrates (Smith and Keinath 2015) (supplementary fig. S1*E* and *F*, Supplementary Material Online). In gene trees, we thus allowed for the possibility of one or two duplications at the base of vertebrates. If two rounds of whole-genome duplication really occurred, this means that we required ohnologs of at least one event to be retained.

We first selected subtrees with a basal speciation node dated at the Chordata taxonomical level—or at the Bilateria taxonomical level when there was no chordate node in the subtree. We classified mouse genes as confident 2R duplicates if the child node of the root of the subtree was a high confidence duplication node dated at the Vertebrata taxonomic level, followed by an optional second high confidence duplication node dated at the Vertebrata taxonomic level, followed by two speciation nodes dated at the Vertebrata taxonomic level, each delineating a subtree containing only one mouse gene and including genes from at least two different fish species. We used Euteleostomi instead of Vertebrata to date the 2R duplications if there was no lamprey gene in the subtree. We classified mouse genes as confident 2R singletons if the child node of the root of the subtree was a speciation node dated at the Vertebrata/Euteleostomi taxonomic level, and delineated a single subtree including one mouse gene and genes from at least two different fish species. We could not enforce strictly the constraint that no duplication occurred in the tetrapod lineage on the branches leading to mouse, because Ensembl mammalian trees include a high number of dubious duplication nodes (duplication confidence score = 0) that are generated when the gene tree topology is not consistent with the species tree. Given the high number of mammalian species in Ensembl, this problem occurred in virtually each of the trees we examined. In total, we obtained 1,389 2R ohnologs and 2,999 singletons.

#### Small-Scale Duplications

We observed that genome assembly and annotation errors resulted in a high number of likely artifactual species-specific paralogs in gene trees (supplementary fig. S1*G* and *H*, Supplementary Material Online). Thus we chose to retain only small-scale duplicates that originated before the split with at least one species. For zebrafish the more recently diverged sister species present in Ensembl was the cave fish *Astyanax mexicanus*, so we focused on small-scale duplicates that originated on the Otophysa branch (deeper branches could not be considered because of the 3R fish-specific genome duplication). We first selected subtrees with a basal speciation node dated at the Clupeocephala taxonomical level. We then retrieved homology relationships between all zebrafish paralogous genes in the subtree (if any), and retained only the high-confidence ones, which did not involve paralogs with 100% sequence identity (probable assembly artifacts) or <10% sequence identity (probable gene split), and were dated at the Otophysa taxonomical level. In total we obtained 385 duplicates.

For mouse we focused on mammal-specific small-scale duplications. We first selected subtrees with a basal speciation node dated at the Mammalia taxonomical level. We then retrieved homology relationships between all mouse paralogous genes in the subtree (if any), and retained only the high-confidence ones, which did not involve paralogs with 100% sequence identity or <10% sequence identity, and were dated at the Theria, Eutheria, Boreoeutheria, Euarchontoglires, Glires, Rodentia, Sciurognathi, or Murinae taxonomical levels. In total, we obtained 646 duplicates.

### Ontology Enrichment Analyses

Enrichment and depletion of expression in anatomical structures were tested with a Fisher exact test using a modified version of the R Bioconductor package topGO (http://bioconductor.org/; Alexa A, personal communications; Gentleman etal. 2004; Alexa etal. 2006; R Development Core Team 2007), allowing to handle other ontologies than the Gene Ontology. We defined the reference set as all the genes for which we had expression data in at least one structure of the organism across all life stages using in situ hybridization data. This accounted for 9,398 genes in zebrafish and 11,322 genes in mouse, expressed in respectively 1,067 and 2,783 anatomical structures. Only anatomical structures with annotated expression of at least five genes were analyzed.

The expression data were propagated to parent structures in the ontology (e.g., a gene expressed in the “hindbrain” was also considered expressed in the parent structure “brain”), a methodology that is very helpful to automatically integrate large amounts of implicit knowledge. However, this can result in the enrichment of nonindependent terms, and of top-level terms of the ontology that are sometimes difficult to interpret, a behavior that is well known for Gene Ontology enrichment tests (Alexa etal. 2006; Falcon and Gentleman 2007; Yon Rhee etal. 2008). To correct for this effect, we used the “weight” algorithm available in the topGO package, a bottom-up approach that up or down-weights terms depending on whether they benefit from the signal of their children structures (Alexa etal. 2006). Unless explicitly mentioned, this algorithm was used in the paper. Using another decorrelation algorithm of the topGO package, the “elim” algorithm, gave similar results (not shown).

A FDR correction was applied on the list of *P*-values from tests on all anatomical structures. Structures enriched or depleted with a FDR < 10% are reported (Benjamini and Hochberg 1995). Of note, all analyses in this paper are reproducible using the TopAnat webservice available at http://bgee.org/?page=top_anat#/, as well as programmatically, using the BgeeDB Bioconductor package available at http://bioconductor.org/packages/release/bioc/html/BgeeDB.html. An example script is available as Supplementary material and on our GitHub repository (file expression_enrichment_with_BgeeDB.R). The results from the webservice and the Bioconductor package can differ slightly from our results due to slight differences on the handling of anatomical ontologies.

### List of Nervous System Anatomical Structures

A reference list of anatomical structures belonging to the nervous system in zebrafish and mouse was extracted from the Uberon ontology (as used in the Bgee database release 13). Because it was sometimes debatable whether a structure belonged to nervous system or not (e.g., sensory organs), we created a “strict” list and a “broad” list.

In zebrafish, the strict list included the “nervous system” structure (UBERON:0001016), as well as its substructures in the ontology. The “sensory system” structure (UBERON:0001032) and its substructures were removed. The broad list included them, as well as presumptive neural structures during development and their substructures (future nervous system, UBERON:0016880; neurectoderm, UBERON:0002346) and the structure “neurovascular bundle” and its substructures (UBERON:0016630).

In mouse, we used the same criteria, but we also noticed that some structures added to Uberon from the mouse-specific ontologies (EMAPA and MA ontologies) were not connected to any nervous system Uberon term at time of study. We thus added the following list of structures and their substructures to our broad list: nerves of urethra (EMAPA:31569), head or neck nerve or ganglion (MA:0000572 and MA:0000580), nerve of prostatic urethra (EMAPA:32279), nerves of urogenital sinus (EMAPA:31533), tail nervous system (EMAPA:16753), testicular branch of genital nerve (EMAPA:29731), nerve of prostate gland (EMAPA:32285), renal cortical nerves (EMAPA:31319), renal medullary nerves (EMAPA:31354), nerve of bladder (EMAPA:31526), nerve of pelvic urethra (EMAPA:31558), and nerve of caudal urethra (EMAPA:31557). Note that many of these species-specific structures are connected to Uberon nervous system structures in the most recent release of Uberon.

The reference lists of nervous system structures were intersected with the list of anatomical structures showing expression of at least five genes, to keep only structures for which expression enrichment was effectively tested.

### List of Anatomical Structures from Other Systems

We selected the high-level terms in the ontologies corresponding to these broad anatomical systems on zebrafish and mouse: Biliary system (UBERON:0002294), Circulatory system (UBERON:0001009), Digestive system (UBERON:0001007), Exocrine system (UBERON:0002330), Hematopoietic system (UBERON:0002390), Immune system (UBERON:0002405), Musculoskeletal (system UBERON:0002204), Renal system (UBERON:0001008), Reproductive system (UBERON:0000990), Respiratory system (UBERON:0001004), and Skeletal system (UBERON:0001434). We then retrieved all the substructures under these high-level terms down to the leaves of the ontology. We randomly picked five terms of the final lists of structures to verify manually that they indeed corresponded to the appropriate anatomical systems. We did not find any false positives during this process.

Similarly to the lists of nervous system structures, we retained in these lists only anatomical structures showing expression of at least five genes.

### Rate of Sequence Evolution

We retrieved the rate of nonsynonymous substitutions *d*_N_ and the rate of synonymous substitutions *d*_S_ for mouse genes from Ensembl release 79 (Hubbard etal. 2009) using BioMart (Smedley etal. 2009). The *d*_N_ and *d*_S_ values were calculated pairwise using one-to-one orthologs in rat (see http://www.ensembl.org/info/genome/compara/homology_method.html#dnds).

### Gene Ontology

We retrieved genes annotated to the Gene Ontology category “metabolic process” (GO:0008152) and its subcategories from the UniProtKB/Swiss-Prot database (The UniProt Consortium 2015), using the following URL: http://www.uniprot.org/uniprot/?query=reviewed:yes+organism:%22Mus%20musculus%20(Mouse)%2010090]%22+go:8152 (queried on August 2, 2016). We performed a similar query to retrieve genes annotated to the category “membrane” (GO:0016020; supplementary text, Supplementary Material online).

### Protein Complexes

We obtained the precise annotation of number of subunits in protein complexes from manually curated information in the UniprotKB/Swiss-Prot database (The UniProt Consortium 2015). We downloaded data on July 21, 2016 using the following URL: http://ebi4.uniprot.org/uniprot/?sort=&desc= &compress=no&query=&fil=reviewed:yes AND organism: “Mus musculus (Mouse) [10090]”&force=no&preview= true&format=tab&columns=id,genes,comment(SUBUNIT). We used regular expressions in a Perl script (available on our GitHub repository) to extract the free-text annotation about involvement in protein complexes in the “SUBUNIT” annotation field. We divided genes into the following categories: monomers (524 genes), homo-multimers (1,936 genes), hetero-dimers (746 genes), hetero-multimers with more than two subunits (e.g., hetero-trimers; 327 genes), and all other complexes that are not described precisely enough to be classified automatically (1,075 genes). The lists of genes in the different categories are available as supplementary material on our GitHub repository (mouse_complexes.zip). If a gene was annotated in multiple categories, we kept it only in the “highest” category, following this order: hetero-multimer > hetero-dimer > uncharacterized complexes > homo-multimers > monomers.

### Connectivity

We retrieved the numbers of direct neighbors of genes in the mouse protein–protein interactions network from the OGEE database. We downloaded the file connectivity.txt.gz at this link: http://ogeedb.embl.de/#download, on July 7, 2016.

### Akashi’s Test

Selection for translational accuracy was tested using Akashi's test (Akashi 1994; Drummond and Wilke 2008), following the procedure described at http://drummond.openwetware.org/Akashi's_Test.html. Alignments of mouse and rat protein-coding genes were retrieved from Ensembl using the Perl API. Sites with the same amino acid at the aligned position in mouse and rat sequences were designated conserved. Optimal codons in mouse were taken from Drummond and Wilke (2008). Laplace smoothing was applied to contingency tables in order to remove problems with counts of zero. The outputs of the test are: (1) a *Z* score, which assesses how likely the association in a gene sequence between conserved sites and preferred codons is to have occurred by chance (significance), and (2) a *Psi* score that assesses how strong is the association between preferred codons and conserved sites, which is computed as an odds ratio.

### Translation Rates

We downloaded the mean of the typical decoding rates (MTDR) index for mouse genes in embryonic stem cells, embryonic fibroblasts and neutrophils from http://www.cs.tau.ac.il/∼tamirtul/MTDR/MTDR_ORF_values/ (Dana and Tuller 2014). The MTDR index represents the geometrical mean of the typical nominal translation rates of codons of a gene, estimated from ribosome profiling data, after filtering biases and the effects of phenomena such as ribosomal traffic jams and translational pauses.

### False Discovery Rates

A FDR of 10% was used to reported anatomical structures showing expression enrichment. For following analyses, where we disentangle the multiple factors associated with duplicate retention rates, we did not find a convenient way to correct for multiple testing. When enough independent tests of similar nature are performed, it is possible to estimate FDRs, but all our tests are not independent. Nonetheless, to give a rough estimate of the FDR in these analyses, we collected all *P*-values generated for the linear regressions of the bin analyses in this paper (51 *P*-values). There was a clear excess of small *P*-values among them, indicating the presence of genuine signal (supplementary fig. S15, Supplementary Material online). Using this list of *P*-values, we estimated that at a *P*-value threshold of 5%, the FDR was well-controlled, at 10.2% using the FDR method (Benjamini and Hochberg 1995), or 3.4% using the *q*-value method (Storey and Tibshirani 2003).

## Supplementary Material

Supplementary data are available at *Molecular Biology and Evolution* online.

## Author Contributions

J.R. made the original observation; J.R. and J.L. designed the detailed study with input from M.R.R.; J.R. and J.L. analyzed the data; J.R. wrote the paper with input from J.L. and M.R.R.

## Supplementary Material

Supplementary Data
