# Supplementary material for: Selective Constraints on Coding Sequences of Nervous System Genes Are a Major Determinant of Duplicate Gene Retention in Vertebrates
Source: Mol Biol Evol. 2017 Jul 16;34(11):2773–91. doi: 10.1093/molbev/msx199 (PMC5850798; doi:10.1093/molbev/msx199)
Supplement: Supplementary Data [file msx199_supp.zip › msx199_SuppFig.pdf]

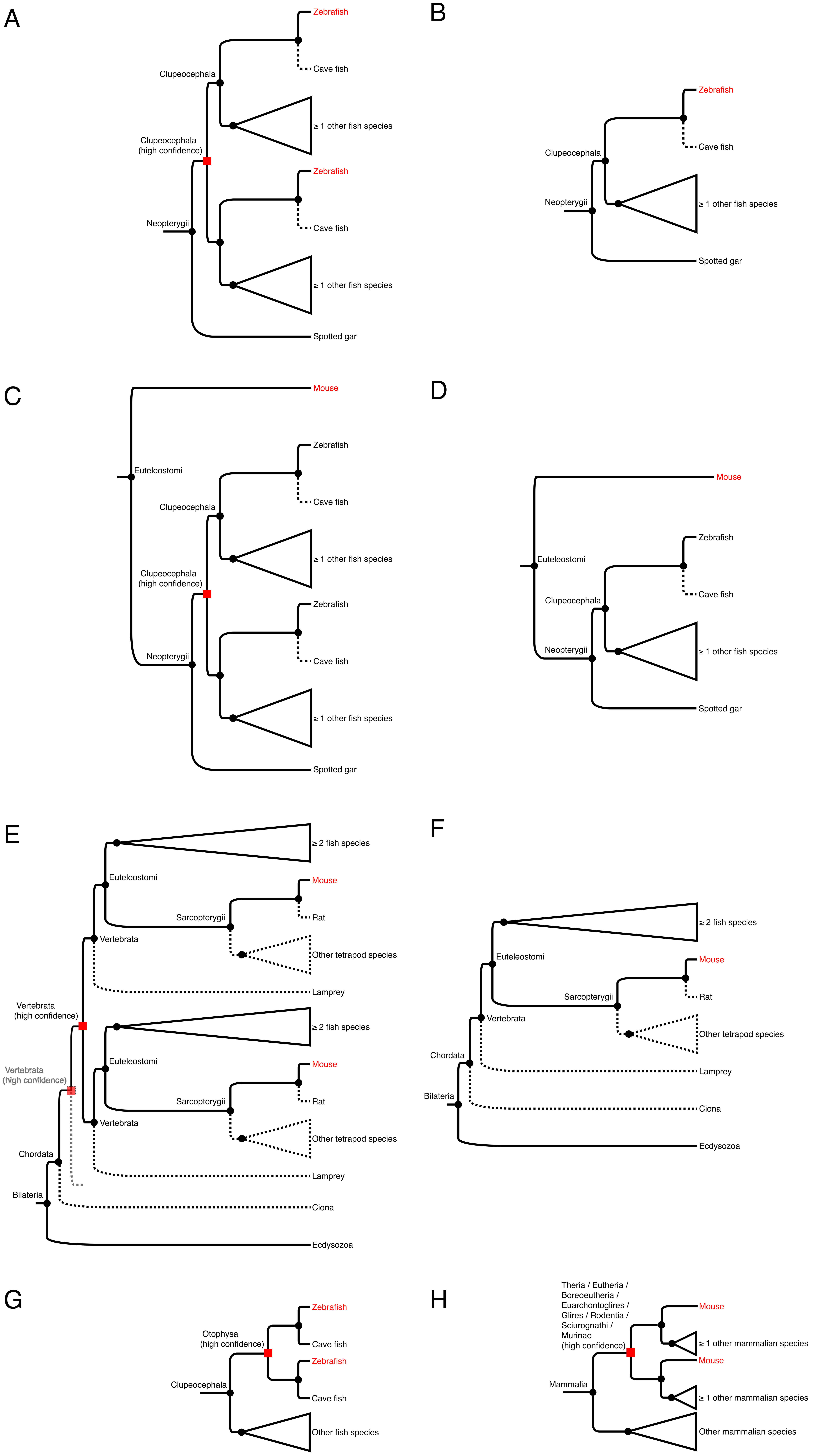

A
Zebrafish
Cave fish
Clupeocephala
≥ 1 other fish species
Clupeocephala
(high confidence)
Zebrafish
Cave fish
Neopterygii
≥ 1 other fish species
Spotted gar
B
Zebrafish
Cave fish
Clupeocephala
Neopterygii
≥ 1 other fish species
Spotted gar
C
Mouse
Zebrafish
Euteleostomi
Cave fish
Clupeocephala
≥ 1 other fish species
Clupeocephala
(high confidence)
Zebrafish
Neopterygii
Cave fish
≥ 1 other fish species
Spotted gar
D
Mouse
Zebrafish
Euteleostomi
Cave fish
Clupeocephala
Neopterygii
≥ 1 other fish species
Spotted gar
E
≥ 2 fish species
Euteleostomi
Mouse
Sarcopterygii
Rat
Vertebrata
Other tetrapod species
Lamprey
Vertebrata
(high confidence)
≥ 2 fish species
Euteleostomi
Mouse
Vertebrata
(high confidence)
Sarcopterygii
Rat
Vertebrata
Other tetrapod species
Chordata
Lamprey
Bilateria
Ciona
Ecdysozoa
F
≥ 2 fish species
Euteleostomi
Mouse
Sarcopterygii
Rat
Vertebrata
Other tetrapod species
Chordata
Lamprey
Bilateria
Ciona
Ecdysozoa
G
Zebrafish
Otophysa
(high confidence)
Cave fish
Zebrafish
Clupeocephala
Cave fish
Other fish species
H
Theria / Eutheria /
Boreoeutheria /
Euarchontoglires /
Glires / Rodentia /
Sciurognathi /
Murinae
(high confidence)
Mouse
≥ 1 other mammalian species
Mouse
Mammalia
≥ 1 other mammalian species
Other mammalian species

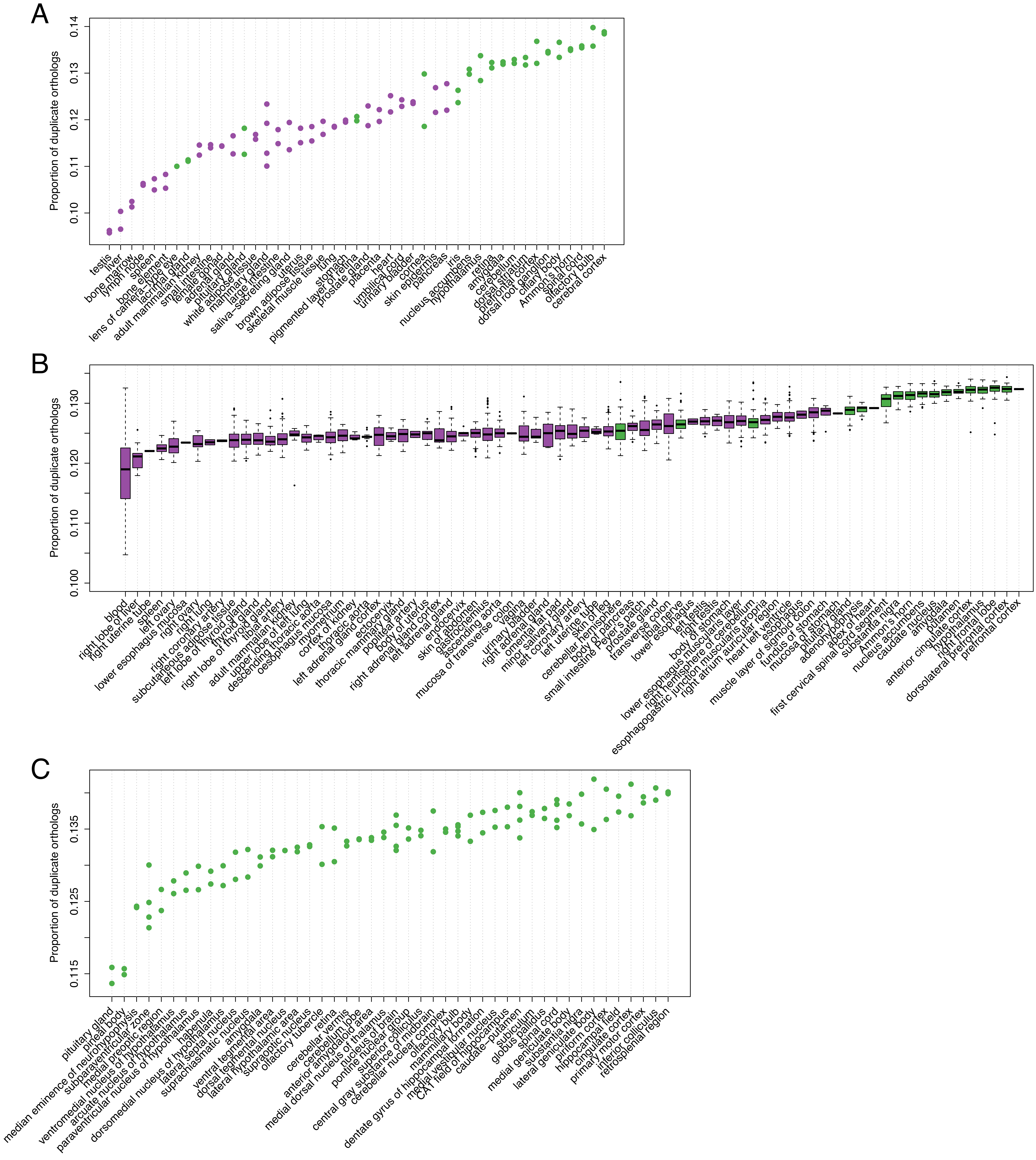
A
Proportion of duplicate orthologs
0.10
0.11
0.12
0.13
0.14
testis
liver
bone marrow
lymph node
spleen
bone element
lens of camera-type eye
lacrimal gland
adult mammalian kidney
small intestine
female gonad
adrenal gland
pituitary gland
white adipose tissue
mammary gland
large intestine
saliva-secreting gland
uterus
brown adipose tissue
skeletal muscle tissue
lung
stomach
pigmented layer of retina
prostate gland
placenta
heart
umbilical cord
urinary bladder
cornea
skin epidermis
pancreas
iris
nucleus accumbens
hypothalamus
retina
amygdala
cerebellum
dorsal striatum
prefrontal cortex
dorsal root ganglion
ciliary body
Ammon's horn
spinal cord
olfactory bulb
cerebral cortex
B
Proportion of duplicate orthologs
0.100
0.110
0.120
0.130
blood
right lobe of liver
right uterine tube
spleen
left ovary
lower esophagus mucosa
right ovary
right lung
right coronary artery
subcutaneous adipose tissue
left lobe of thyroid gland
thyroid gland
right lobe of thyroid gland
tibial artery
adult mammalian kidney
upper lobe of left lung
descending thoracic aorta
oesophagus mucosa
myometrium
cortex of kidney
thoracic aorta
left adrenal gland cortex
ectocervix
thoracic mammary gland
popliteal artery
body of uterus
right adrenal gland cortex
left adrenal gland
endocervix
skin of abdomen
gastrocnemius
ascending aorta
mucosa of transverse colon
vagina
urinary bladder
right adrenal gland
omental fat pad
minor salivary gland
left coronary artery
left uterine tube
skin of leg
cerebellar hemisphere
body of pancreas
small intestine Peyer's patch
prostate gland
transverse colon
tibial nerve
lower esophagus
left testis
right testis
body of stomach
lower esophagus muscularis layer
right hemisphere of cerebellum
esophagogastric junction muscularis propria
right atrium auricular region
heart left ventricle
esophagus
muscle layer of sigmoid colon
fundus of stomach
mucosa of stomach
pituitary gland
adenohypophysis
apex of heart
first cervical spinal cord segment
substantia nigra
Ammon's horn
nucleus accumbens
caudate nucleus
amygdala
putamen
anterior cingulate cortex
hypothalamus
right frontal lobe
dorsolateral prefrontal cortex
prefrontal cortex
C
Proportion of duplicate orthologs
0.115
0.125
0.135
pituitary gland
pineal body
median eminence of neurohypophysis
subparaventricular zone
medial preoptic region
ventromedial nucleus of hypothalamus
arcuate nucleus of hypothalamus
paraventricular nucleus of hypothalamus
habenula
dorsomedial nucleus of hypothalamus
lateral septal nucleus
suprachiasmatic nucleus
amygdala
ventral tegmental area
dorsal tegmental nucleus
lateral hypothalamic area
supraoptic nucleus
olfactory tubercle
retina
cerebellar vermis
anterior amygdaloid area
cerebellum lobe
medial dorsal nucleus of thalamus
nucleus of brain
pontine nuclear group
superior colliculus
central gray substance of midbrain
cerebellar nuclear complex
olfactory bulb
dentate gyrus of hippocampal formation
mammillary body
medial vestibular nucleus
CA1 field of hippocampus
caudate-putamen
subiculum
globus pallidus
spinal cord
medial geniculate body
substantia nigra
lateral geniculate body
piriform cortex
hippocampal field
cingulate cortex
primary motor cortex
inferior colliculus
retrosplenial region

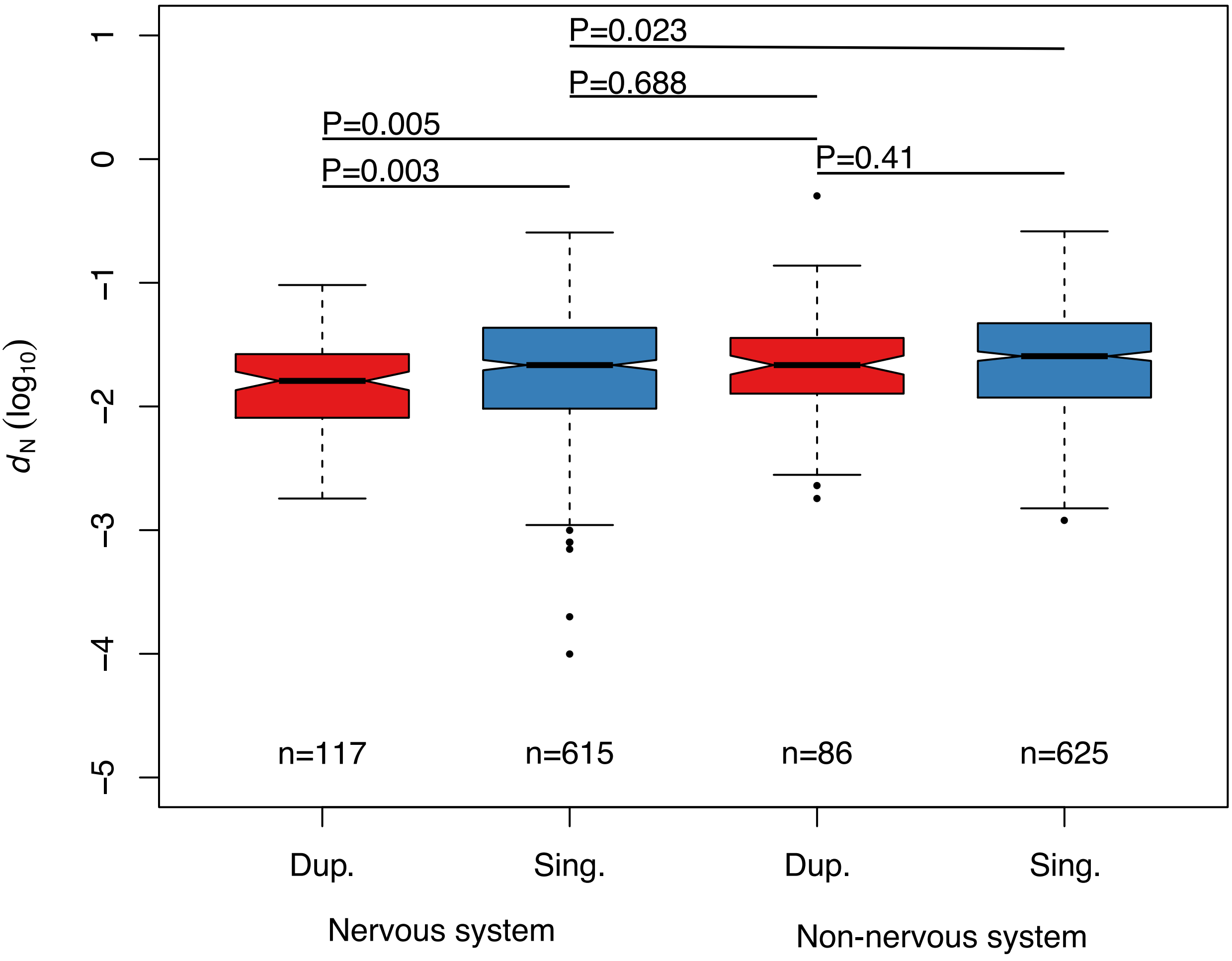

P=0.023
P=0.688
P=0.005
P=0.003
P=0.41
d_N (log10)
1
0
−1
−2
−3
−4
−5
n=117
n=615
n=86
n=625
Dup.
Sing.
Dup.
Sing.
Nervous system
Non-nervous system

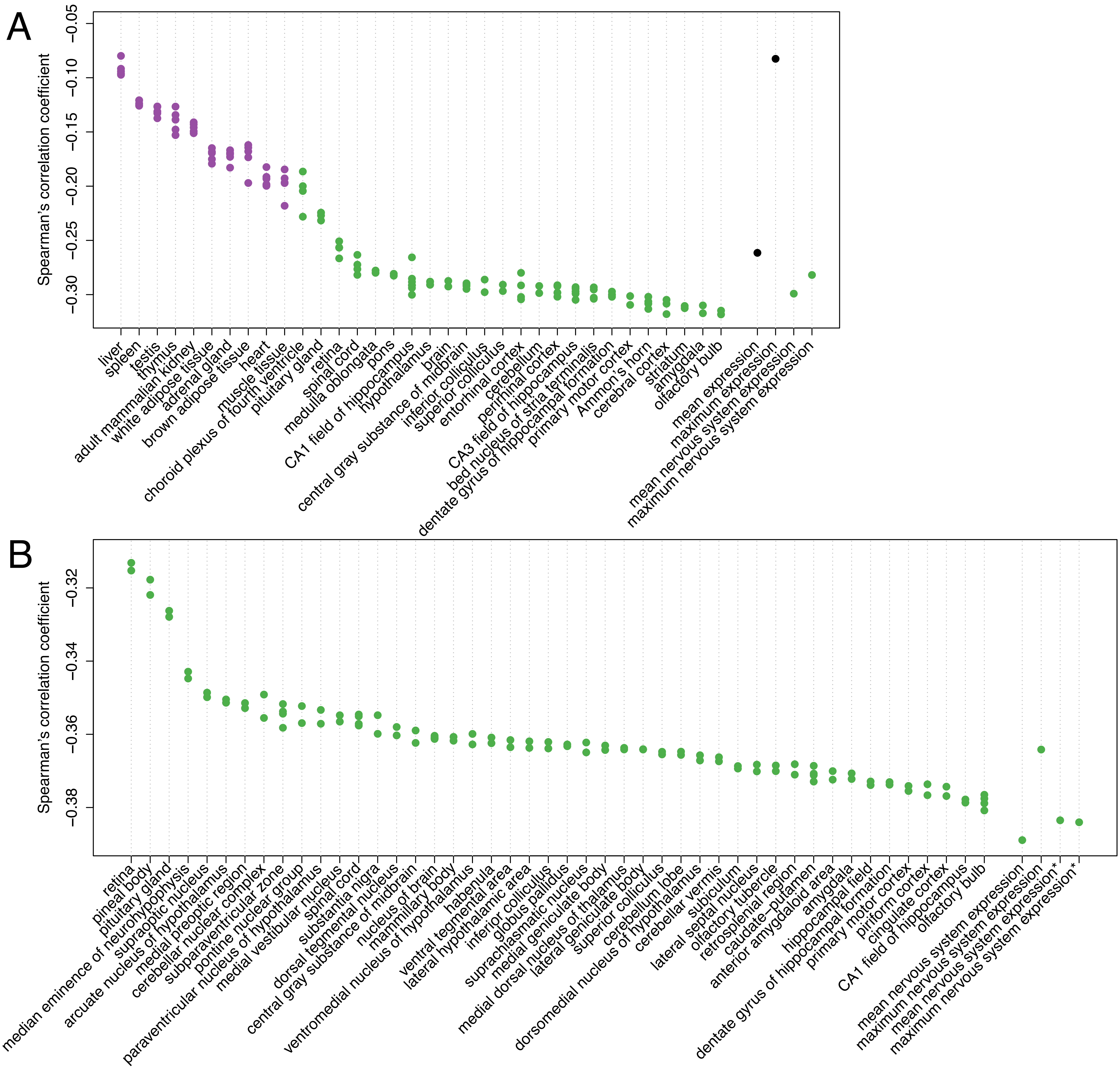
A
Spearman's correlation coefficient
−0.05
−0.10
−0.15
−0.20
−0.25
−0.30
liver
spleen
testis
thymus
adult mammalian kidney
white adipose tissue
adrenal gland
brown adipose tissue
heart
muscle tissue
choroid plexus of fourth ventricle
pituitary gland
retina
spinal cord
medulla oblongata
pons
CA1 field of hippocampus
hypothalamus
central gray substance of midbrain
brain
inferior colliculus
superior colliculus
entorhinal cortex
cerebellum
CA3 field of hippocampus
perirhinal cortex
bed nucleus of stria terminalis
dentate gyrus of hippocampal formation
primary motor cortex
Ammon's horn
cerebral cortex
striatum
amygdala
olfactory bulb
mean expression
maximum expression
mean nervous system expression
maximum nervous system expression
B
Spearman's correlation coefficient
−0.32
−0.34
−0.36
−0.38
retina
pineal body
pituitary gland
median eminence of neurohypophysis
supraoptic nucleus
arcuate nucleus of hypothalamus
medial preoptic region
cerebellar nuclear complex
subparaventricular zone
pontine nuclear group
paraventricular nucleus of hypothalamus
medial vestibular nucleus
spinal cord
substantia nigra
dorsal tegmental nucleus
central gray substance of midbrain
nucleus of brain
mammillary body
ventromedial nucleus of hypothalamus
habenula
ventral tegmental area
lateral hypothalamic area
inferior colliculus
globus pallidus
suprachiasmatic nucleus
medial geniculate body
medial dorsal nucleus of thalamus
lateral geniculate body
superior colliculus
cerebellum lobe
dorsomedial nucleus of hypothalamus
cerebellar vermis
subiculum
lateral septal nucleus
olfactory tubercle
retrosplenial region
caudate−putamen
anterior amygdaloid area
amygdala
hippocampal field
dentate gyrus of hippocampal formation
primary motor cortex
piriform cortex
cingulate cortex
CA1 field of hippocampus
olfactory bulb
mean nervous system expression
maximum nervous system expression
mean nervous system expression*
maximum nervous system expression*

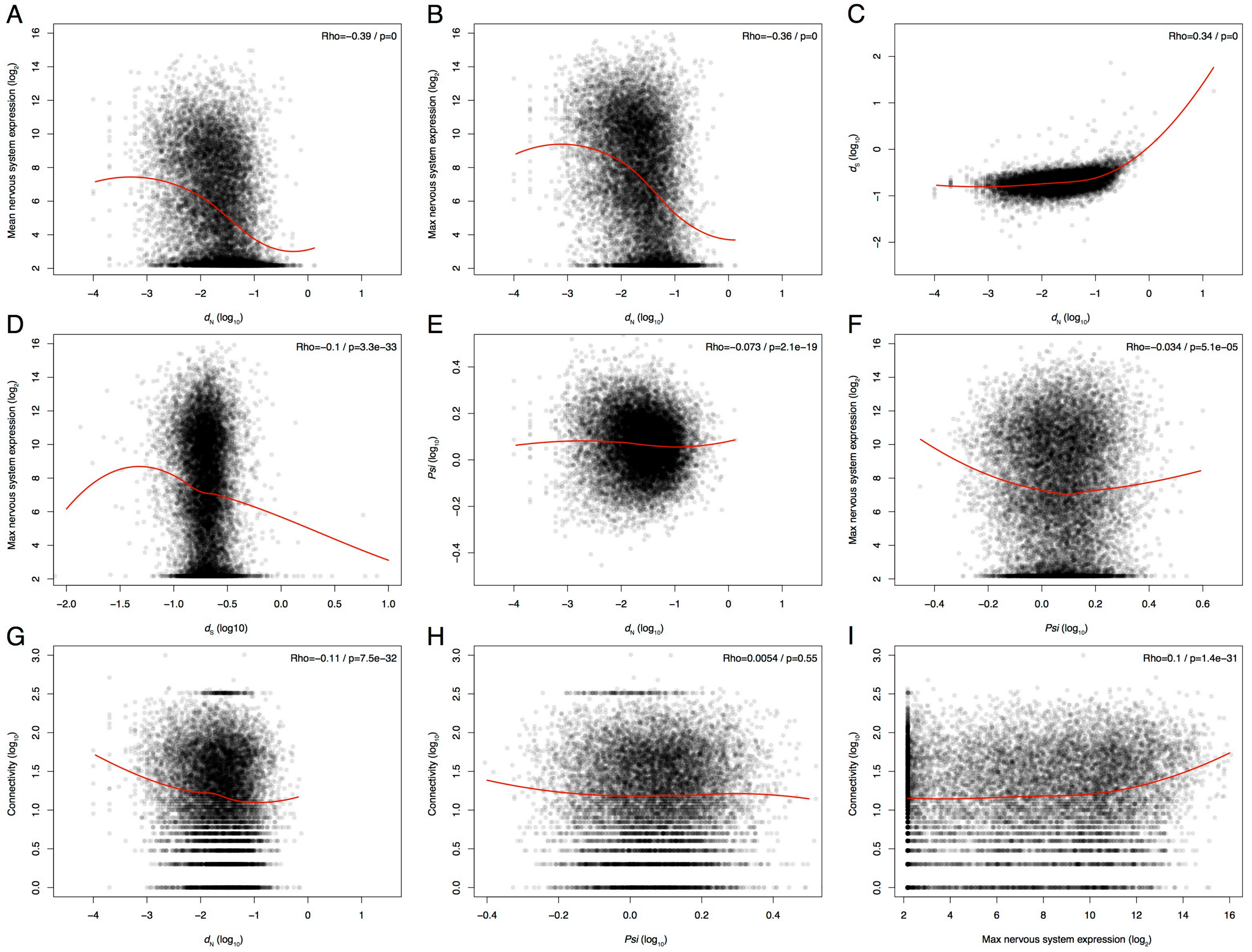
A
Rho=−0.39 / p=0
Mean nervous system expression (log2)
dN (log10)
B
Rho=−0.36 / p=0
Max nervous system expression (log2)
dN (log10)
C
Rho=0.34 / p=0
dS (log10)
dN (log10)
D
Rho=−0.1 / p=3.3e−33
Max nervous system expression (log2)
dS (log10)
E
Rho=−0.073 / p=2.1e−19
Psi (log10)
dN (log10)
F
Rho=−0.034 / p=5.1e−05
Max nervous system expression (log2)
Psi (log10)
G
Rho=−0.11 / p=7.5e−32
Connectivity (log10)
dN (log10)
H
Rho=0.0054 / p=0.55
Connectivity (log10)
Psi (log10)
I
Rho=0.1 / p=1.4e−31
Connectivity (log10)
Max nervous system expression (log2)

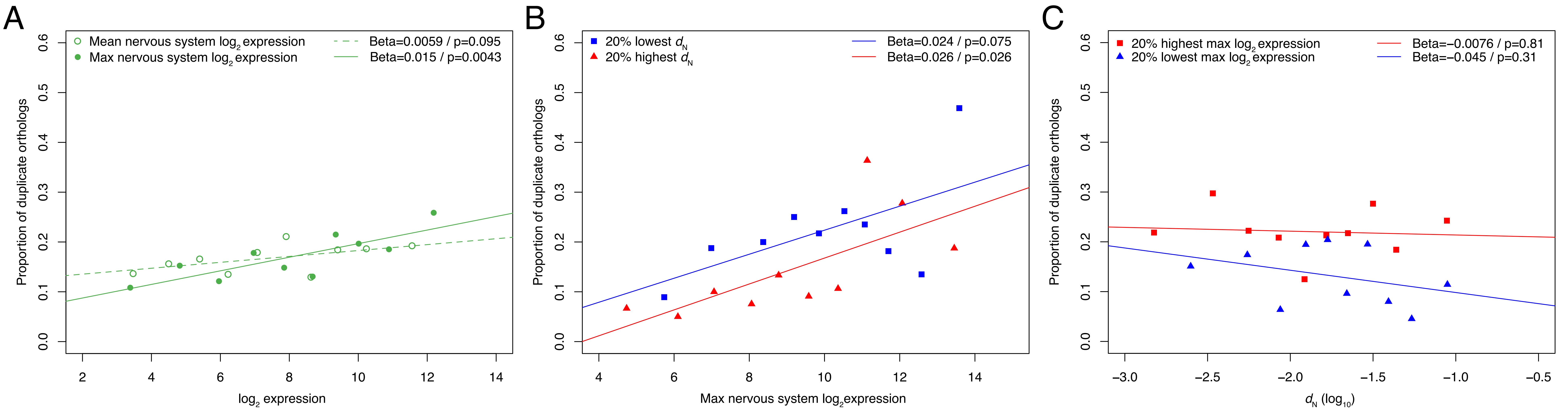

A
Mean nervous system log2 expression
Max nervous system log2 expression
Beta=0.0059 / p=0.095
Beta=0.015 / p=0.0043
Proportion of duplicate orthologs
log2 expression
B
20% lowest dN
20% highest dN
Beta=0.024 / p=0.075
Beta=0.026 / p=0.026
Max nervous system log2expression
C
20% highest max log2 expression
20% lowest max log2 expression
Beta=−0.0076 / p=0.81
Beta=−0.045 / p=0.31
dN (log10)

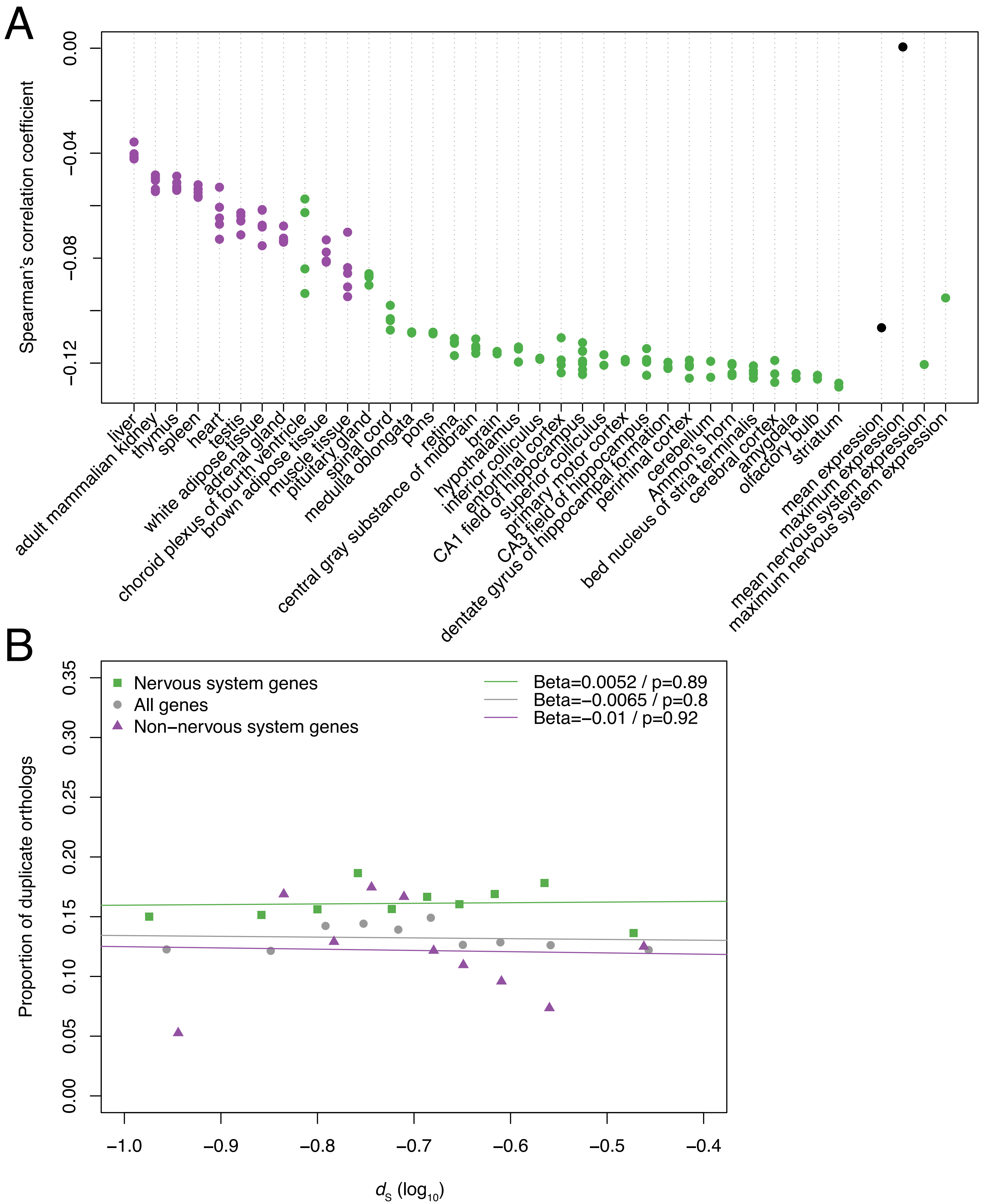

A
Spearman's correlation coefficient
0.00
−0.04
−0.08
−0.12
liver
adult mammalian kidney
thymus
spleen
heart
testis
white adipose tissue
adrenal gland
choroid plexus of fourth ventricle
brown adipose tissue
muscle tissue
pituitary gland
spinal cord
medulla oblongata
pons
central gray substance of midbrain
retina
brain
hypothalamus
inferior colliculus
entorhinal cortex
CA1 field of hippocampus
superior colliculus
primary motor cortex
CA3 field of hippocampus
dentate gyrus of hippocampal formation
perirhinal cortex
cerebellum
Ammon's horn
bed nucleus of stria terminalis
cerebral cortex
amygdala
olfactory bulb
striatum
mean expression
maximum expression
mean nervous system expression
maximum nervous system expression
B
Nervous system genes
All genes
Non−nervous system genes
Beta=0.0052 / p=0.89
Beta=−0.0065 / p=0.8
Beta=−0.01 / p=0.92
Proportion of duplicate orthologs
0.00
0.05
0.10
0.15
0.20
0.25
0.30
0.35
−1.0
−0.9
−0.8
−0.7
−0.6
−0.5
−0.4
d_S (log_{10})

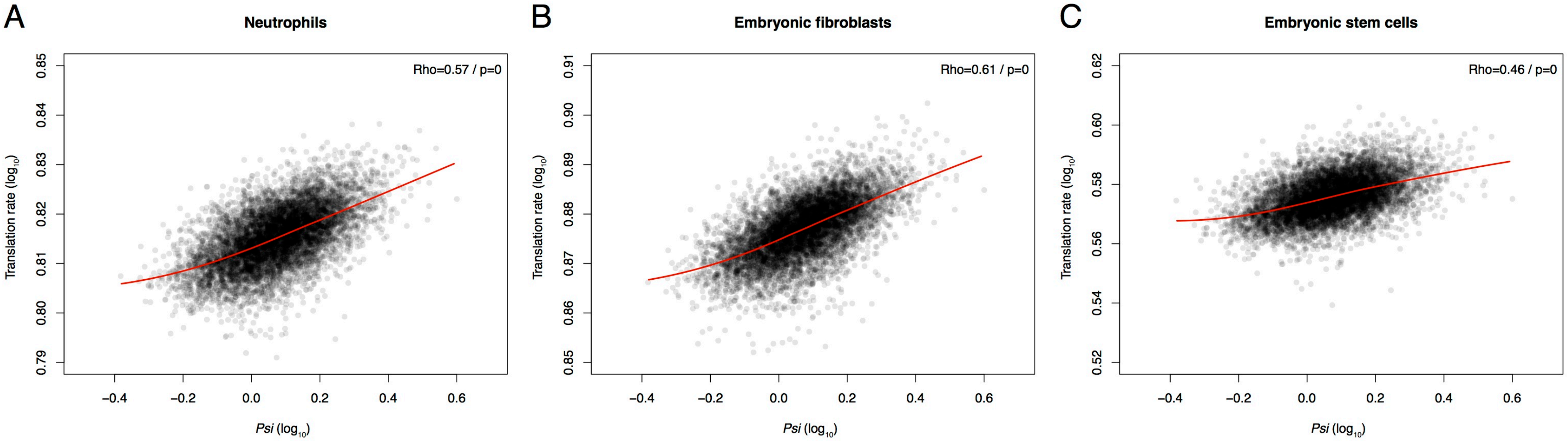

A
Neutrophils
Rho=0.57 / p=0
Translation rate (log10)
Psi (log10)
B
Embryonic fibroblasts
Rho=0.61 / p=0
Translation rate (log10)
Psi (log10)
C
Embryonic stem cells
Rho=0.46 / p=0
Translation rate (log10)
Psi (log10)

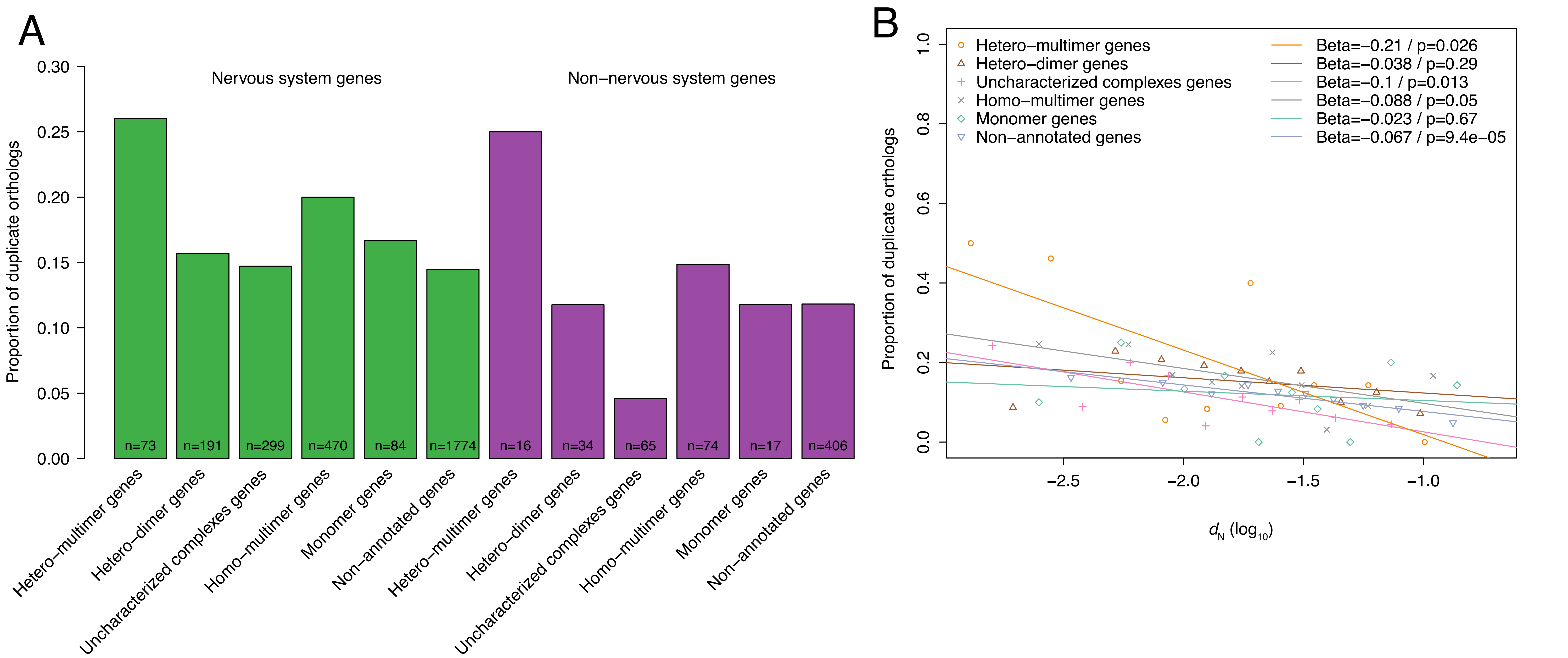

A
Nervous system genes
Non–nervous system genes
Proportion of duplicate orthologs
0.30
0.25
0.20
0.15
0.10
0.05
0.00
n=73
n=191
n=299
n=470
n=84
n=1774
n=16
n=34
n=65
n=74
n=17
n=406
Hetero–multimer genes
Hetero–dimer genes
Uncharacterized complexes genes
Homo–multimer genes
Monomer genes
Non–annotated genes
B
Hetero–multimer genes
Hetero–dimer genes
Uncharacterized complexes genes
Homo–multimer genes
Monomer genes
Non–annotated genes
Beta=–0.21 / p=0.026
Beta=–0.038 / p=0.29
Beta=–0.1 / p=0.013
Beta=–0.088 / p=0.05
Beta=–0.023 / p=0.67
Beta=–0.067 / p=9.4e–05
1.0
0.8
0.6
0.4
0.2
0.0
–2.5
–2.0
–1.5
–1.0
d_N ($\log_{10}$)

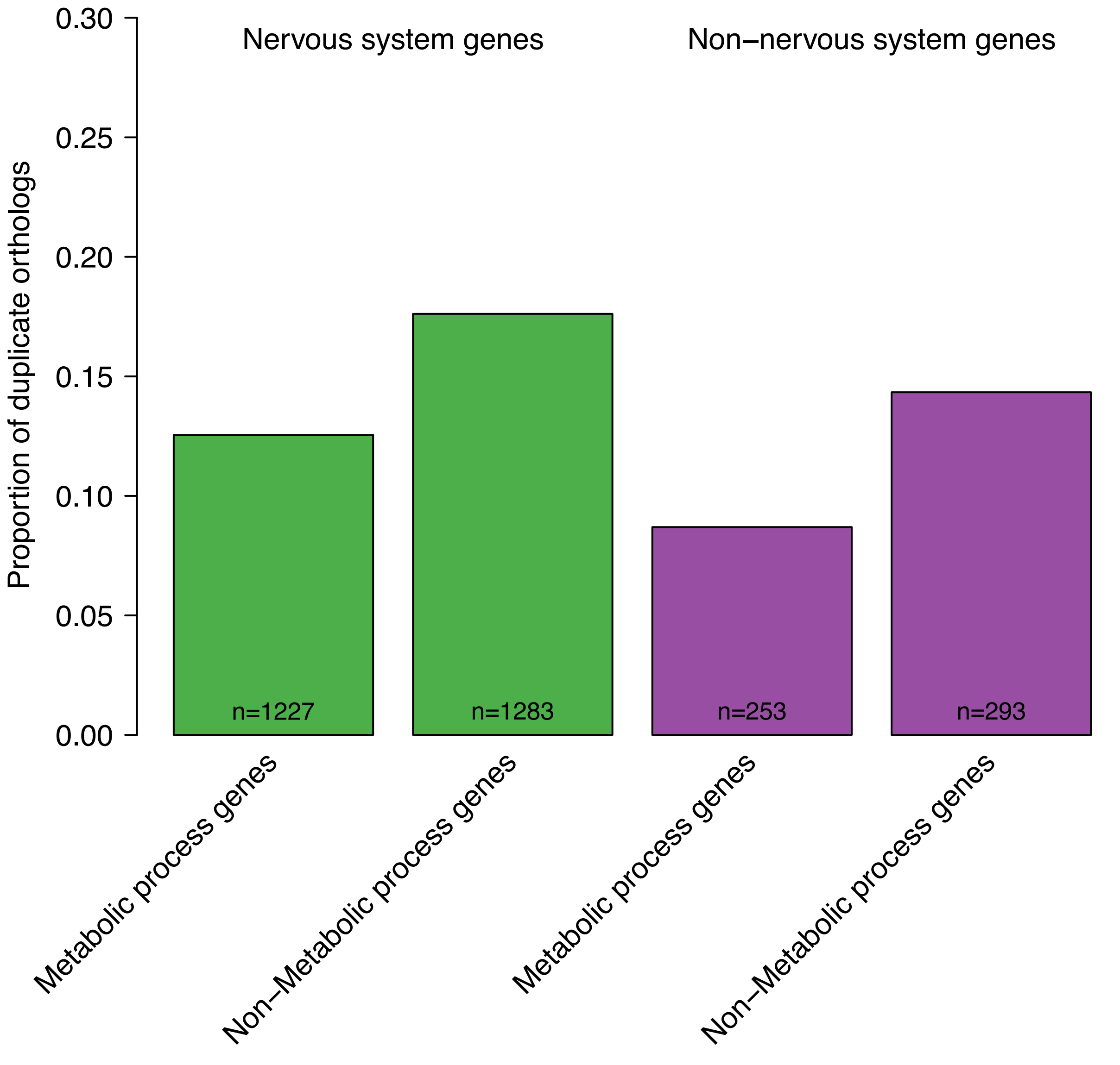

Nervous system genes
Non−nervous system genes
Proportion of duplicate orthologs
0.30
0.25
0.20
0.15
0.10
0.05
0.00
n=1227
n=1283
n=253
n=293
Metabolic process genes
Non−Metabolic process genes
Metabolic process genes
Non−Metabolic process genes

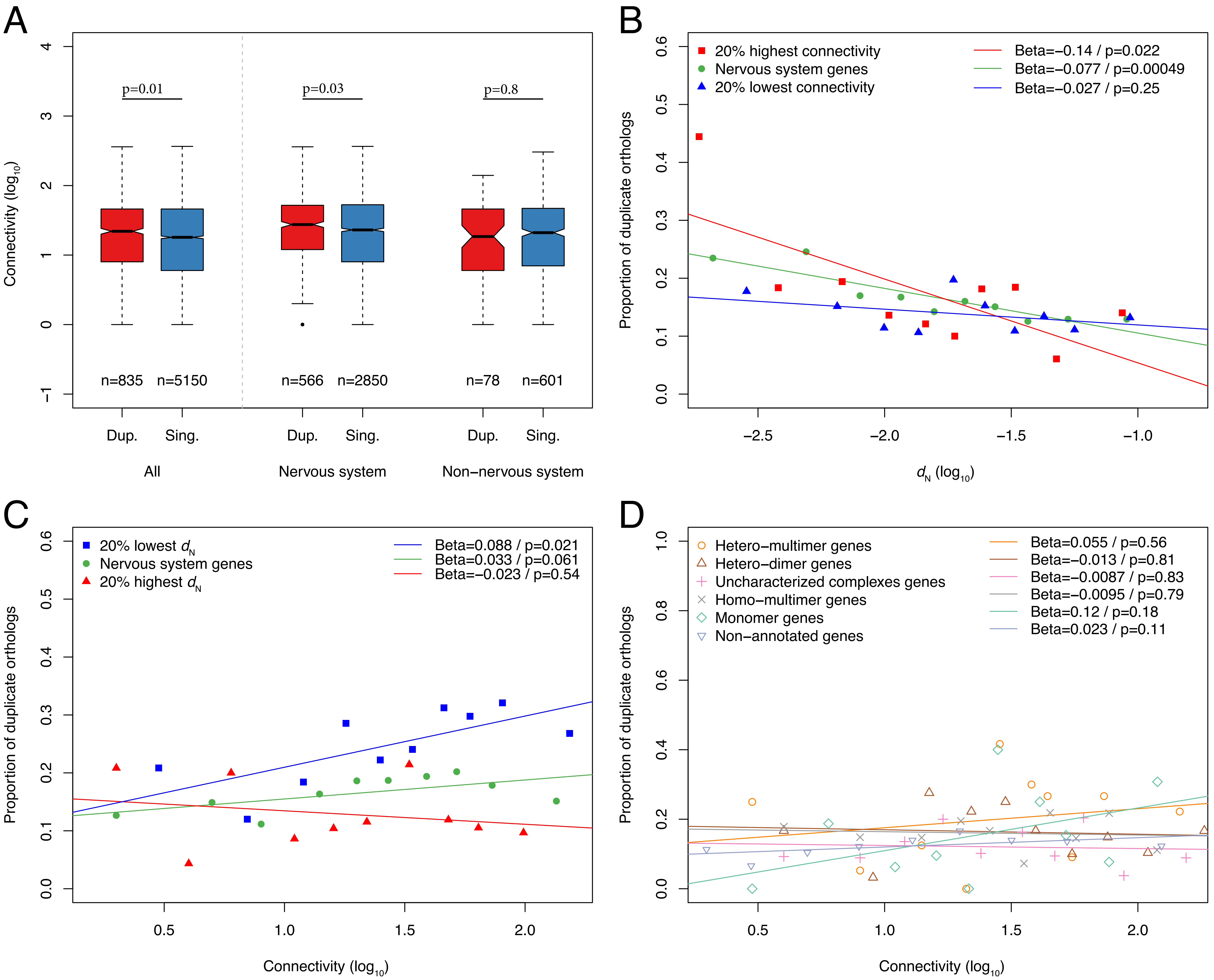
A
Connectivity (log10)
p=0.01
p=0.03
p=0.8
n=835 n=5150
n=566 n=2850
n=78 n=601
Dup. Sing. Dup. Sing. Dup. Sing.
All
Nervous system
Non−nervous system
B
Proportion of duplicate orthologs
20% highest connectivity
Nervous system genes
20% lowest connectivity
Beta=−0.14 / p=0.022
Beta=−0.077 / p=0.00049
Beta=−0.027 / p=0.25
dN (log10)
C
Proportion of duplicate orthologs
20% lowest dN
Nervous system genes
20% highest dN
Beta=0.088 / p=0.021
Beta=0.033 / p=0.061
Beta=−0.023 / p=0.54
Connectivity (log10)
D
Proportion of duplicate orthologs
Hetero−multimer genes
Hetero−dimer genes
Uncharacterized complexes genes
Homo−multimer genes
Monomer genes
Non−annotated genes
Beta=0.055 / p=0.56
Beta=−0.013 / p=0.81
Beta=−0.0087 / p=0.83
Beta=−0.0095 / p=0.79
Beta=0.12 / p=0.18
Beta=0.023 / p=0.11
Connectivity (log10)

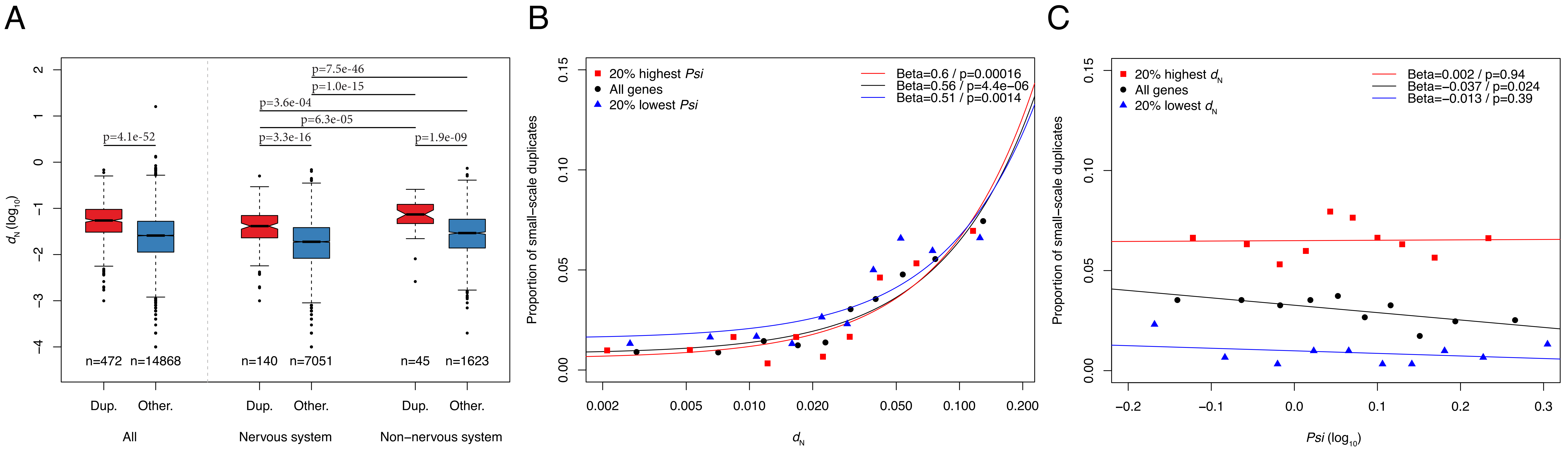

A
B
C
p=7.5e-46
p=1.0e-15
p=3.6e-04
p=6.3e-05
p=4.1e-52
p=3.3e-16
p=1.9e-09
d_N ($\log_{10}$)
n=472
n=14868
n=140
n=7051
n=45
n=1623
Dup.
Other.
All
Nervous system
Non−nervous system
20% highest *Psi*
All genes
20% lowest *Psi*
Beta=0.6 / p=0.00016
Beta=0.56 / p=4.4e−06
Beta=0.51 / p=0.0014
Proportion of small−scale duplicates
d_N
20% highest d_N
20% lowest d_N
Beta=0.002 / p=0.94
Beta=−0.037 / p=0.024
Beta=−0.013 / p=0.39
Psi ($\log_{10}$)

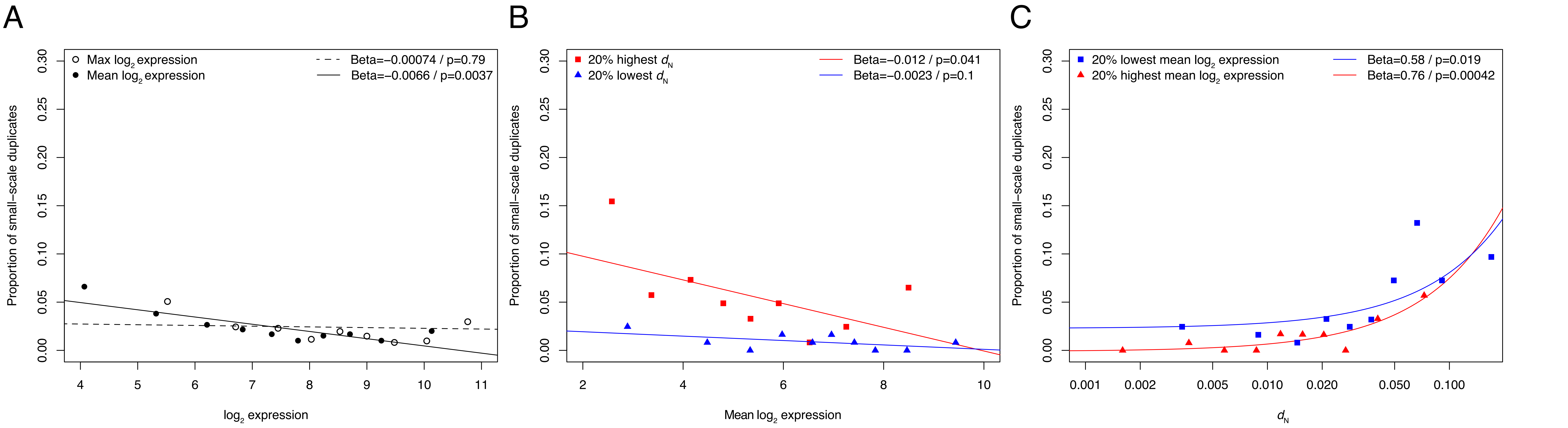
A
B
C
Max log2 expression
Mean log2 expression
Beta=−0.00074 / p=0.79
Beta=−0.0066 / p=0.0037
Proportion of small−scale duplicates
log2 expression
20% highest dN
20% lowest dN
Beta=−0.012 / p=0.041
Beta=−0.0023 / p=0.1
Mean log2 expression
20% lowest mean log2 expression
20% highest mean log2 expression
Beta=0.58 / p=0.019
Beta=0.76 / p=0.00042
dN

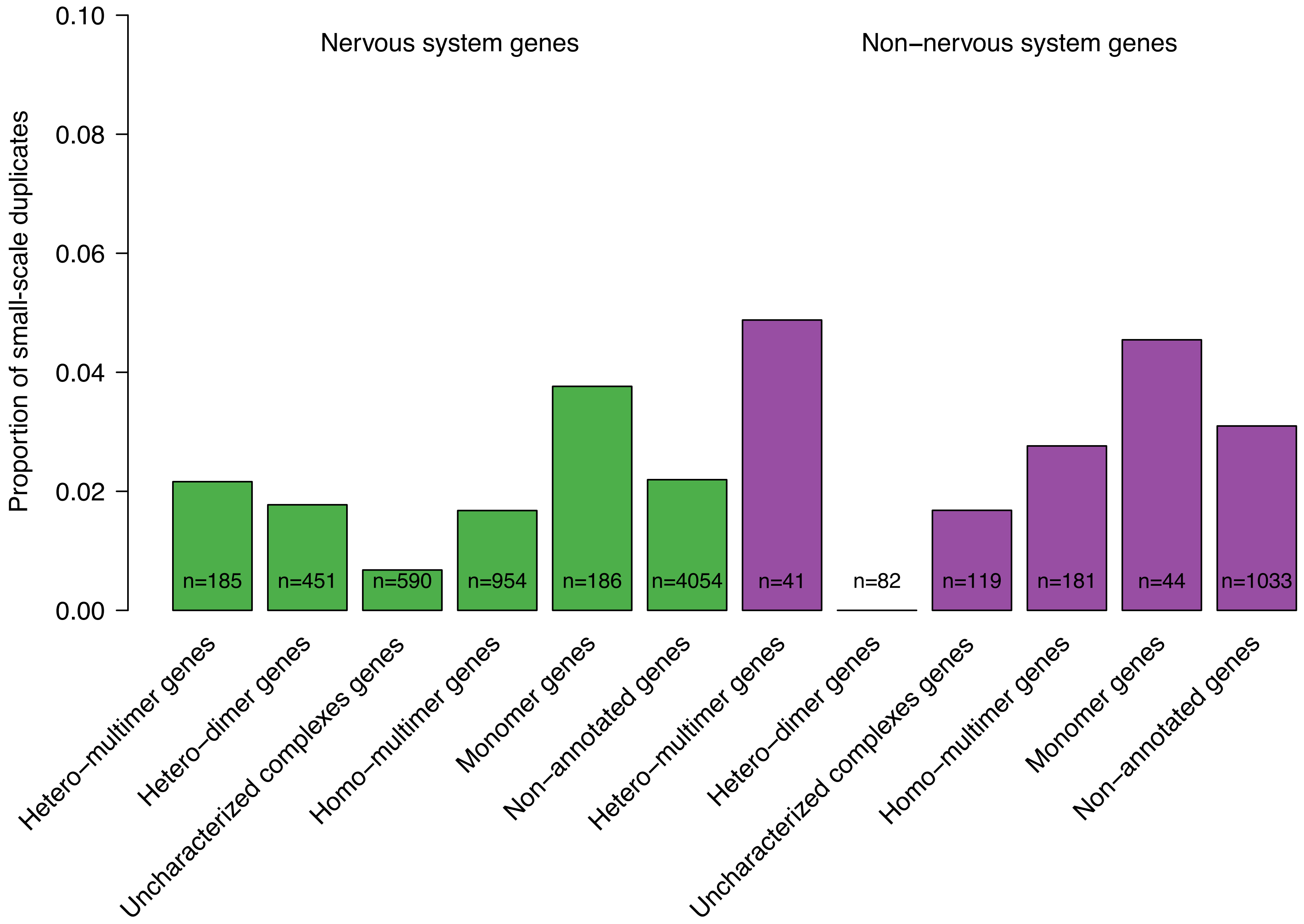

Nervous system genes
Non−nervous system genes
Proportion of small-scale duplicates
0.10
0.08
0.06
0.04
0.02
0.00
n=185
n=451
n=590
n=954
n=186
n=4054
n=41
n=82
n=119
n=181
n=44
n=1033
Hetero−multimer genes
Hetero−dimer genes
Uncharacterized complexes genes
Homo−multimer genes
Monomer genes
Non−annotated genes
Hetero−multimer genes
Hetero−dimer genes
Uncharacterized complexes genes
Homo−multimer genes
Monomer genes
Non−annotated genes

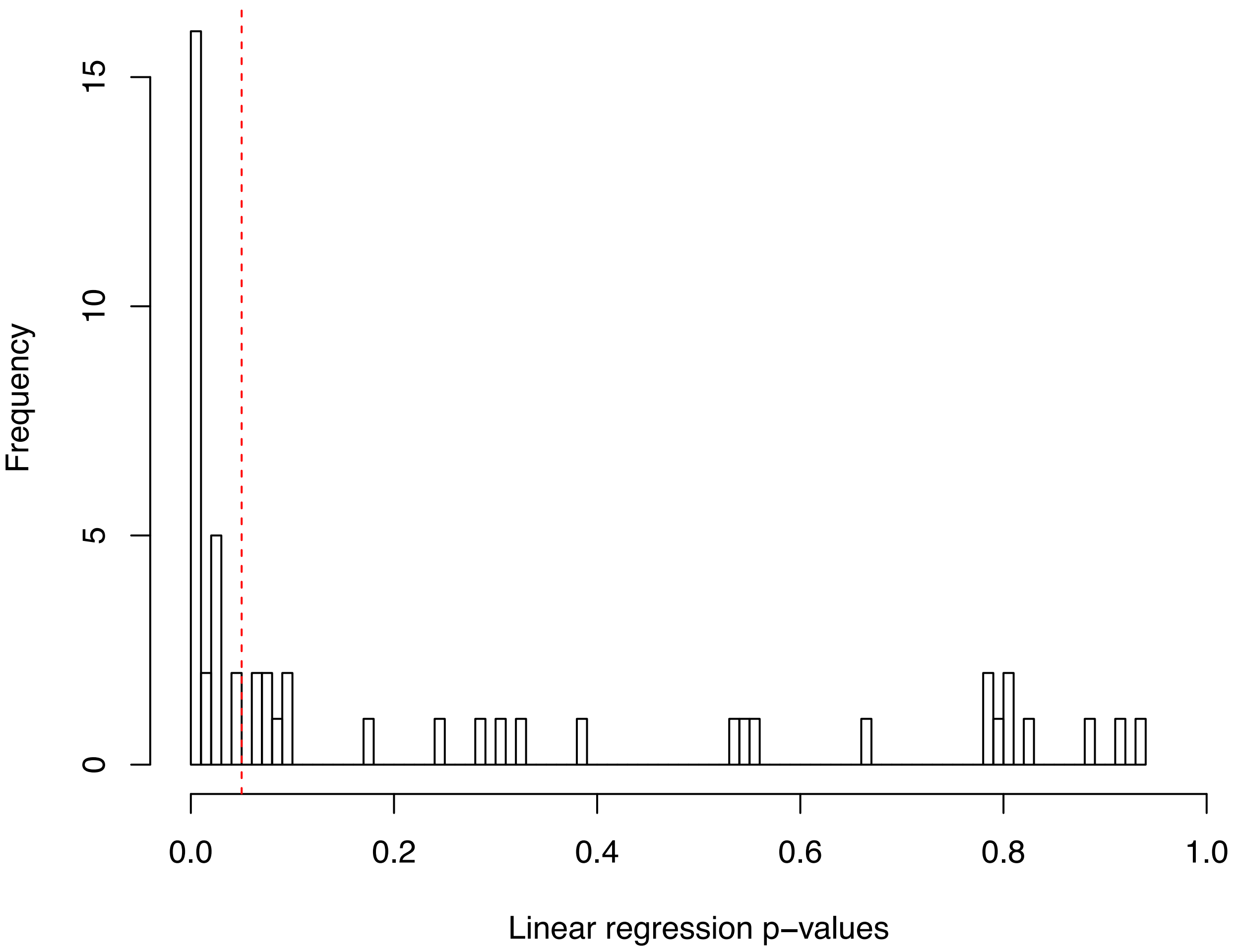
15
10
5
0
Frequency
0.0
0.2
0.4
0.6
0.8
1.0
Linear regression p−values

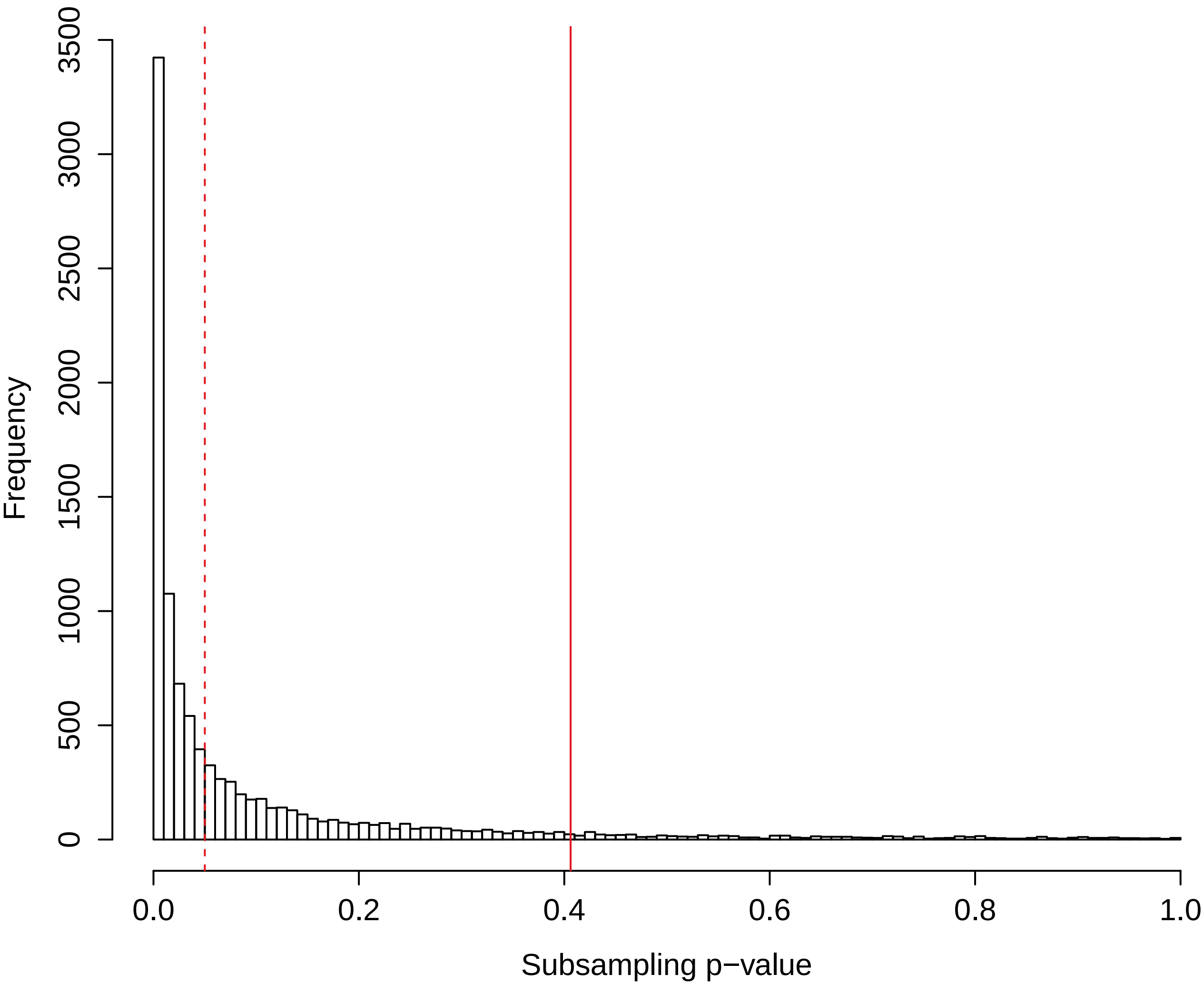

Frequency
0
500
1000
1500
2000
2500
3000
3500
0.0
0.2
0.4
0.6
0.8
1.0
Subsampling p−value

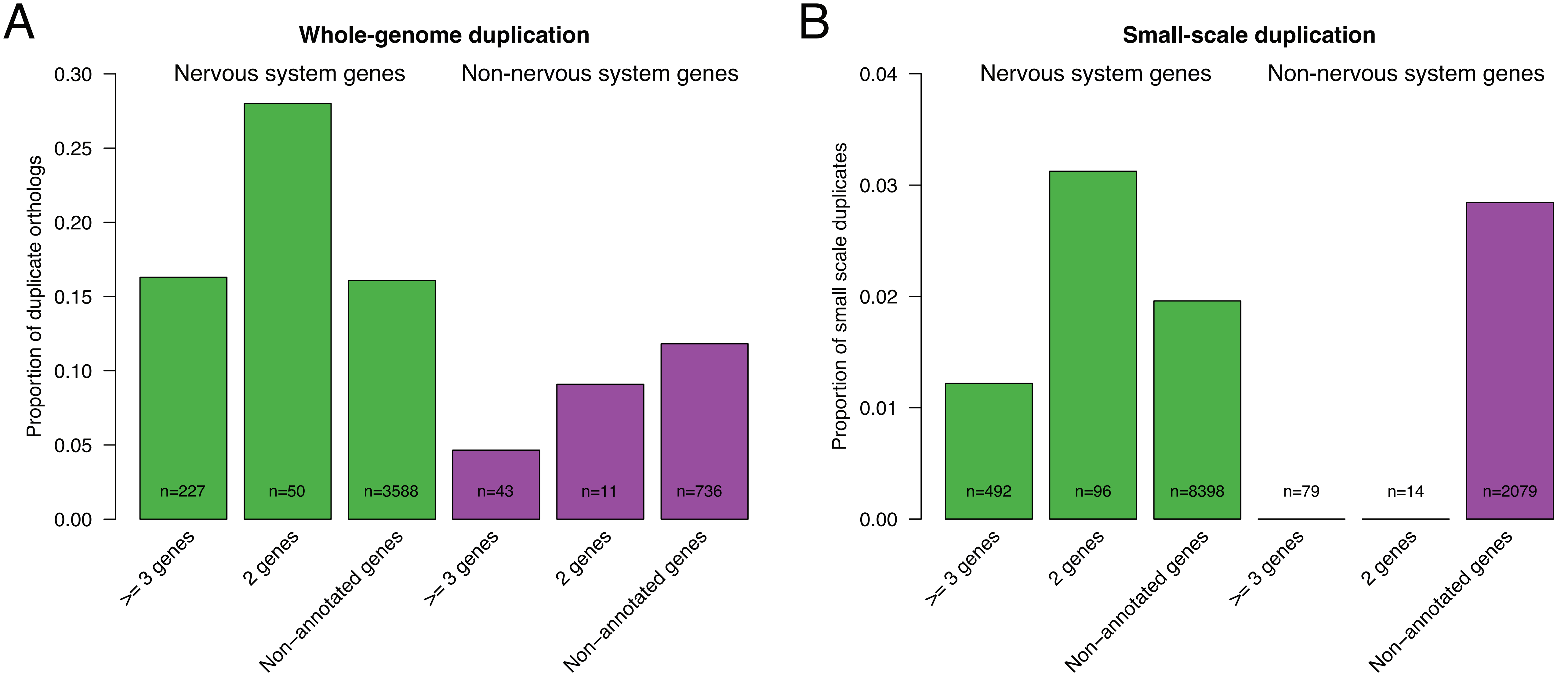
A
Whole-genome duplication
Nervous system genes
Non-nervous system genes
Proportion of duplicate orthologs
0.30
0.25
0.20
0.15
0.10
0.05
0.00
n=227
n=50
n=3588
n=43
n=11
n=736
>= 3 genes
2 genes
Non–annotated genes
>= 3 genes
2 genes
Non–annotated genes
B
Small-scale duplication
Nervous system genes
Non-nervous system genes
Proportion of small scale duplicates
0.04
0.03
0.02
0.01
0.00
n=492
n=96
n=8398
n=79
n=14
n=2079
>= 3 genes
2 genes
Non–annotated genes
>= 3 genes
2 genes
Non–annotated genes

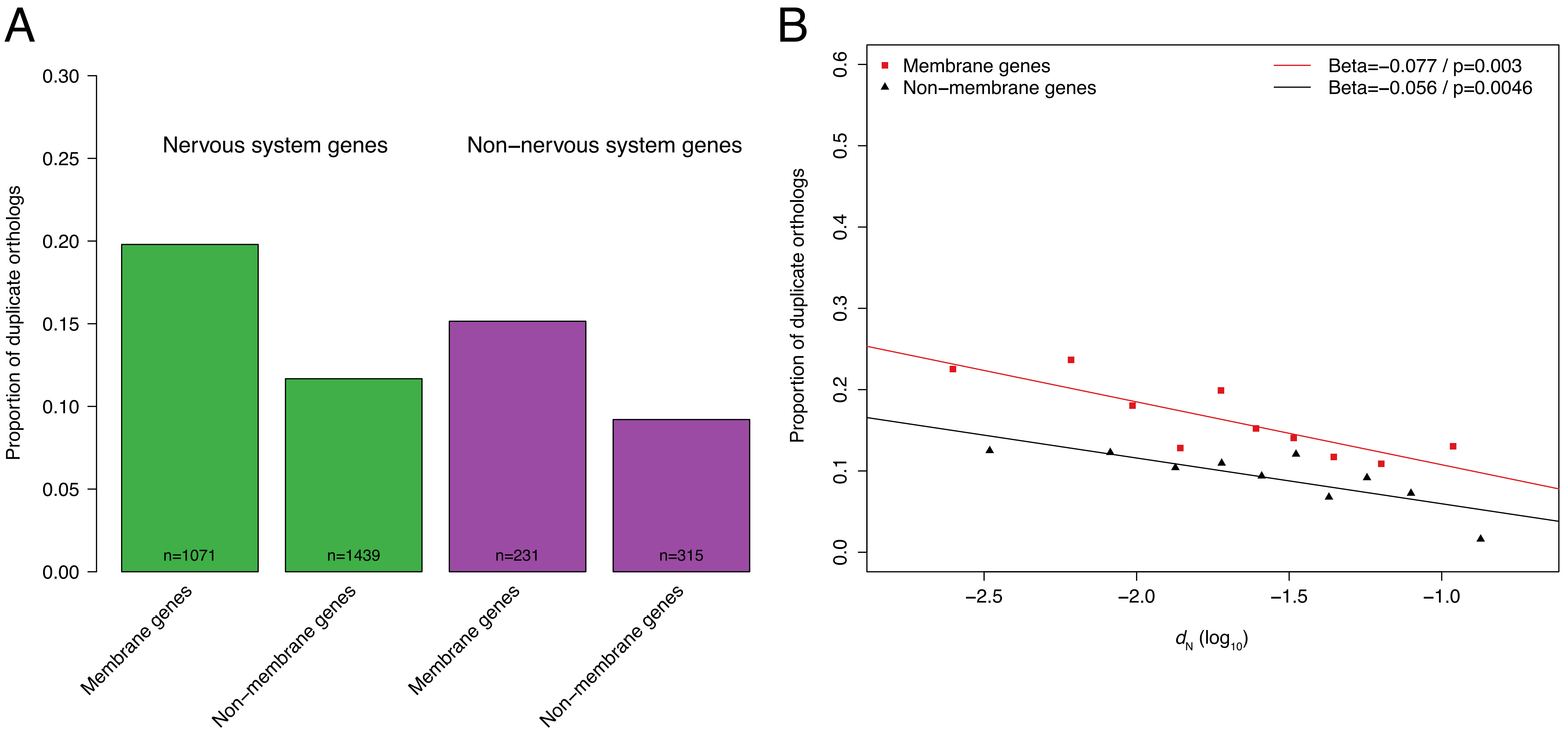
A
Nervous system genes
Non−nervous system genes
Proportion of duplicate orthologs
0.30
0.25
0.20
0.15
0.10
0.05
0.00
n=1071
n=1439
n=231
n=315
Membrane genes
Non−membrane genes
Membrane genes
Non−membrane genes
B
Membrane genes
Non−membrane genes
Beta=−0.077 / p=0.003
Beta=−0.056 / p=0.0046
Proportion of duplicate orthologs
0.6
0.5
0.4
0.3
0.2
0.1
0.0
−2.5
−2.0
−1.5
−1.0
d_N ($\log_{10}$)

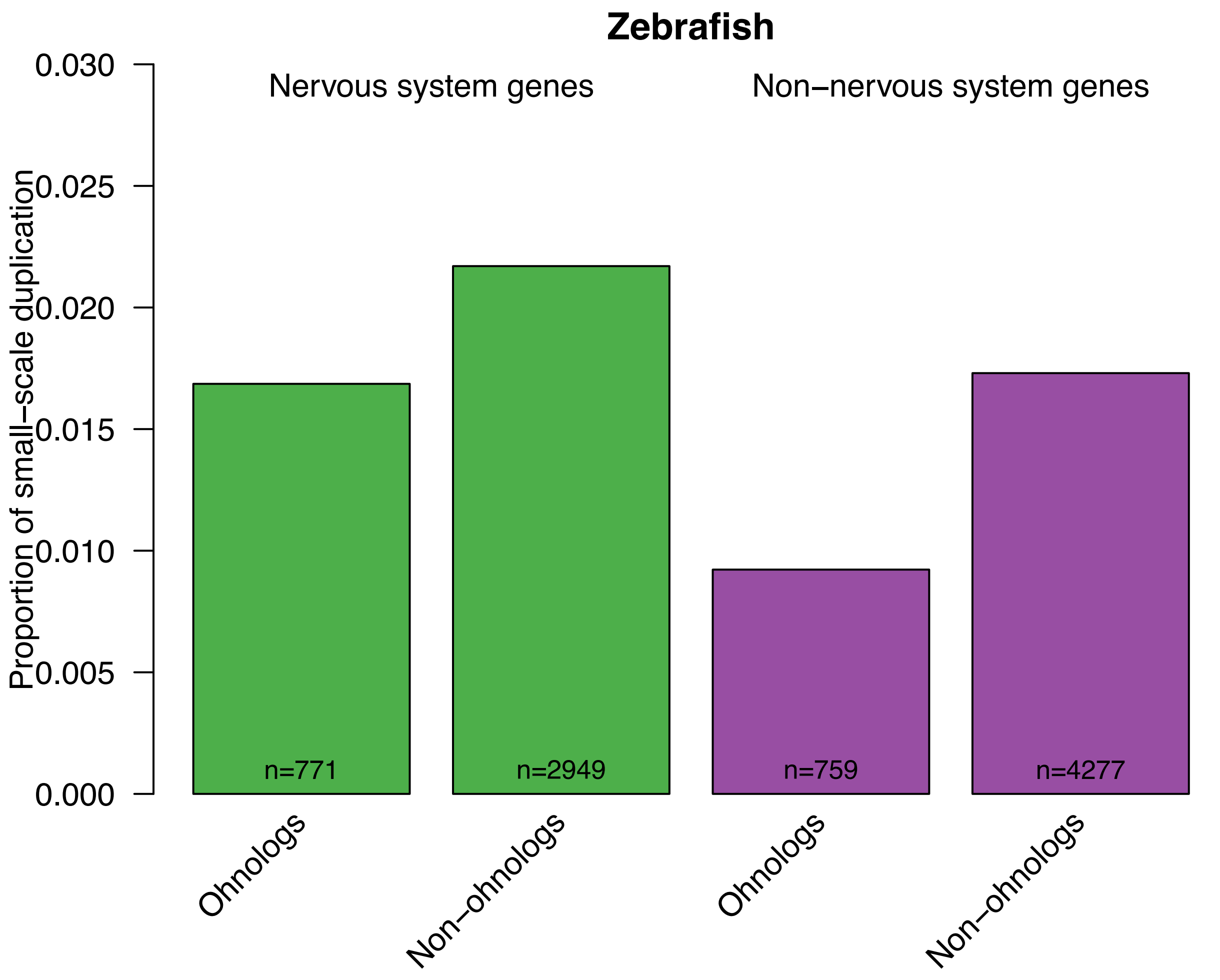

Zebrafish
Nervous system genes
Non−nervous system genes
Proportion of small−scale duplication
0.030
0.025
0.020
0.015
0.010
0.005
0.000
n=771
n=2949
n=759
n=4277
Ohnologs
Non−ohnologs
Ohnologs
Non−ohnologs
