# Supplementary material for: Selective Constraints on Coding Sequences of Nervous System Genes Are a Major Determinant of Duplicate Gene Retention in Vertebrates
Source: Mol Biol Evol. 2017 Jul 16;34(11):2773–91. doi: 10.1093/molbev/msx199 (PMC5850798; doi:10.1093/molbev/msx199)
Supplement: Supplementary Data [file msx199_supp.zip › msx199_Supptext.doc]

Supplementary text

# Selective constraints on coding sequences of nervous system genes are a major determinant of duplicate gene retention in vertebrates

Julien Roux, Jialin Liu, Marc Robinson-Rechavi

## Power issues in *d*_N_ comparisons

When sub-dividing mouse orthologs of zebrafish 3R ohnologs and orthologs of singletons (Figure 2B) based on their expression in the nervous system, a significantly slower rate of evolution of orthologs of ohnologs was observed for nervous system genes (p=5.31e-08), but not for non-nervous system genes (p=0.41). Since the number of number of nervous system genes included in this analysis is higher than the number non-nervous system genes, the high p-value observed for the latter could be due to a lower power of the test.

To address this, we subsampled nervous system genes to obtain a sample size similar to non-nervous system genes. We randomly subsampled 86 nervous system orthologs of ohnologs, and 625 nervous system orthologs of singletons (10,000 times) and looked at the distribution of p-values from the Wilcoxon tests between the log_10_(*d*_N_) of duplicates and singletons (Figure S16). Out of the 10,000 p-values, 9,345 were lower than p=0.41, and 6,109 were lower than p=0.05. This result indicated that the difference in significance between nervous system genes comparisons and non-nervous system genes comparisons did not likely reflect power issues.

## Test of the dosage-balance hypothesis with CORUM data

Similarly to the analysis with UniProt data, we also tested the dosage-balance hypothesis using protein complex data from the CORUM database (the Comprehensive Resource of Mammalian protein complexes, accessible at http://mips.helmholtz-muenchen.de/genre/proj/corum/). Dosage imbalance should impact the formation of complexes involving more than two genes. Complexes involving two genes should be impacted if they include more than two subunits, but this information is not available in CORUM. This category thus includes a mix of complexes more or less sensitive to dosage. Finally, very few monomers or homo-multimers are annotated in CORUM, so we used all non-annotated genes as reference.

We mapped Entrez gene IDs to Ensembl gene IDs and classified genes as members of complexes involving at least three different Entrez IDs (hetero-multimeric; 772 genes), two different Entrez IDs (240 genes), or a unique Entrez ID (homo-multimeric; 17 genes). Since the latter category included very few genes, we did not include it in our analyses. If a gene was mapped to different complexes, involving a different number of genes, we kept it in the category involving the highest number of genes, probably the most sensitive to dosage balance . Genes were split into nervous system and non-nervous system genes.

Surprisingly, while dosage sensitivity should be the strongest for members of complexes involving at least three genes, the proportion of mouse orthologs of zebrafish 3R duplicates was lower than in other categories, both for nervous and non-nervous system genes (Figure S17A).

Regarding small-scale duplicates, complexes involving more than two genes displayed the lowest proportion of small-scale duplicates, a result consistent with the dosage-balance hypothesis (Figure S17B).

**Membrane proteins are more retained after whole-genome duplication**

It was previously shown in *Paramecium* that membrane proteins tend to be the least retained in duplicate after whole-genome duplication. This pattern was explained by the decrease of the cell surface to volume ratio after whole-genome duplication, leading to excessive surface concentration of membrane proteins .

Since neurons are very elongated cells, their surface to volume ratio is higher than other cell types. After whole-genome duplication the surface concentration of membrane proteins could thus increase in lower proportion for neurons than for other cell types, potentially explaining the nervous system retention bias.

But we observed no difference between nervous and non-nervous system genes, and moreover the trend was opposite to the *Paramecium* results: the proportion of mouse orthologs of zebrafish 3R duplicates was higher for membrane proteins than non-membrane (Figure S18A).

We did not find any explanation to this surprising result, which was independent of other potential confounding factors (e.g., *d*_N_ see Figure S18B). More analyses are required to understand this pattern, which fall outside of the scope of this paper. A previous studies has suggested that membrane proteins are evolving fast , maybe leading to faster rates of sub- or new-functionalization.

**Small-scale duplications are not under-represented in nervous system ohnologs**

Although there is no a priori reason to suspect that dosage imbalance effects might be more severe in the nervous system than in other tissues, such a scenario could result in the selective retention of nervous system ohnologs. It should also result in an under-representation of small-scale duplications among nervous system ohnologs compared to non-nervous system ohnologs.

In the data used in the paper, only ohnologs without secondary small-scale duplications were selected, so we retrieved a less stringent set of zebrafish ohnologs without applying this criterion and estimated their propensity to duplicate later in small-scale duplications, compared to the rest of genes (non-ohnologs), taking their nervous system expression patterns into account (Figure S19).

On the one hand, we observed, as expected, that the proportion of small-scale duplications in ohnologs was lower than the proportion of small-scale duplications in non-ohnologs, both for genes expressed in the nervous system or not, although these differences were not significant (p=0.48 and p=0.12 for nervous system and non-nervous system respectively; p=0.14 when both groups are considered together).

On the other hand, we observed surprisingly a higher proportion of small-scale duplicates for ohnologs expressed in the nervous system than for ohnologs not expressed in the nervous system. This difference is not significant either (Fisher exact test, p=0.26), but its direction goes against the idea of an interdependence of the effects of dosage imbalance and the expression of genes in the nervous system (see Discussion).

1. Ruepp A, Waegele B, Lechner M, Brauner B, Dunger-Kaltenbach I, et al. (2010) CORUM: the comprehensive resource of mammalian protein complexes--2009. Nucleic Acids Res 38: D497-501.

2. Veitia RA, Bottani S, Birchler JA (2008) Cellular reactions to gene dosage imbalance: genomic, transcriptomic and proteomic effects. Trends Genet 24: 390-397.

3. Gout J-F, Kahn D, Duret L, Paramecium Post-Genomics C (2010) The Relationship among Gene Expression, the Evolution of Gene Dosage, and the Rate of Protein Evolution. PLoS Genet 6: e1000944.

4. Victor S, Dessimoz C, Pomiankowski A, Lane N (2016) Membrane proteins are dramatically less conserved than water-soluble proteins across the tree of life. Molecular Biology and Evolution.
